# Multifunctional coatings of nickel-titanium implant toward promote osseointegration after operation of bone tumor and clinical application: a review

**DOI:** 10.3389/fbioe.2024.1325707

**Published:** 2024-02-20

**Authors:** Tianhao Du, Jia Liu, Jinhan Dong, Haoxu Xie, Xiao Wang, Xu Yang, Yingxin Yang

**Affiliations:** ^1^ Department of Rehabilitation Medicine, General Hospital of Northern Theater Command, Shenyang, China; ^2^ Liaoning University of traditional Chinese Medicine, Shenyang, China

**Keywords:** nickel-titanium alloy, orthopedic clinical implants, osseointegration, multifunctional coatings, porous nickel titanium alloy

## Abstract

Metal implants, especially Ni-Ti shape memory alloy (Ni-Ti SMA) implants, have increasingly become the first choice for fracture and massive bone defects after orthopedic bone tumor surgery. In this paper, the internal composition and shape memory properties of Ni-Ti shape memory alloy were studied. In addition, the effects of porous Ni-Ti SMA on osseointegration, and the effects of surface hydrophobicity and hydrophilicity on the osseointegration of Ni-Ti implants were also investigated. In addition, the effect of surface coating modification technology of Ni-Ti shape memory alloy on bone bonding was also studied. Several kinds of Ni-Ti alloy implants commonly used in orthopedic clinic and their advantages and disadvantages were introduced. The surface changes of Ni-Ti alloy implants promote bone fusion, enhance the adhesion of red blood cells and platelets, promote local tissue regeneration and fracture healing. In the field of orthopaedics, the use of Ni-Ti shape memory alloy implants significantly promoted clinical development. Due to the introduction of the coating, the osseointegration and biocompatibility of the implant surface have been enhanced, and the success rate of the implant has been greatly improved.

## 1 Introduction

Bone and soft tissue tumors are diseases that seriously endanger human health and life. In recent years, the incidence of primary malignant bone tumors has increased gradually. Primary malignant bone tumors are mostly seen in teenagers and middle-aged people between 10 and 30 years old, including osteosarcoma, Ewing sarcoma, chondrosarcoma, malignant fibrous histiocytoma, chordoma and so on (Sbaraglia et al., 2020). Osteosarcoma (OS), a type of bone tumor that occurs in its original location, accounts for approximately 35% of all primary bone malignancies. There is a male-to-female ratio of about 1.5%. The majority of instances, around 80%–90%, happen in body regions that have elongated tubular epiphyses, such as the hands and feet. Additionally, the distal femur and proximal tibia are frequently affected near the knee joint, whereas the proximal humerus is commonly involved near the shoulder joint. Standard care consists of timely surgical removal, supplementary chemotherapy, immunotherapy, and targeted medication, leading to a rise in the 5-year survival percentage to 60%–70%. However, a significant portion of patients (40%) continue to show poor responses to the prescribed therapy (Meltzer and Helman, 2021; Shi et al., 2023). Adriamycin, danomycin, and deschumab are among the primary medications sanctioned by the Food and Drug Administration (FDA) to treat OS. Additionally, methotrexate, doxorubicin, and cisplatin are commonly employed for this purpose in North America and Europe (Quadros et al., 2021). According to a recent study, it has been forecasted and demonstrated that strophanthidin is a successful medication for managing OS (Chen et al., 2022). In addition to drug therapy, targeted therapy (Wu et al., 2018) and orthopedic implants after OS are also common in clinic.

Since the early 1960s, orthopedic implants have been made of three different types of materials: metal, ceramic and polymer (Yuan et al., 2010; Bao et al., 2017). In orthopaedics, the use of biomaterials such as silicate, stainless steel, titanium, polyether ether ketone (PEEK), zirconia, alumina ceramics, polylactic acid (PLLA), and polyglycolic acid (PGA) has become increasingly prevalent due to the advancements in 3D printing technology in recent years (Xu et al., 2022). Due to its excellent shape memory capabilities and remarkable superelasticity, the utilization of Niti shape memory alloy has been extensive in various sectors including aerospace, metallurgy, and manufacturing. Furthermore, apart from its exceptional malleability, minimal magnetism, ability to resist corrosion and fatigue, as well as its compatibility with living organisms, it possesses various other desirable characteristics. Consequently, it has found extensive application in the realm of medicine (Dennis et al., 2015).

Ni-Ti shape memory alloy is utilized in different internal fixation devices within the medical industry, including shape memory orthopaedic nail, fibula embracing fixator, wrist triangle fusion cage, interbody fusion cage, and others. It is also used to make vascular stents, vascular embolizers, vascular staplers and intestinal staplers. In the field of medicine, shape memory alloys primarily utilize their exceptional superelastic properties. Over the past few years, there has been an increasing focus on the Ni-Ti shape memory alloy, which is now widely acknowledged as an excellent material for implants (Wu et al., 2018). In spinal fusion implants, solid titanium is commonly utilized, and nickel-titanium implants should also be promoted in this kind of surgery (Aihara et al., 2019). To prevent mechanical failure, orthopedic implants require an ideal biomaterial with excellent tensile strength, fatigue strength, a Young’s modulus comparable to bone, and complete resistance to corrosion (Biesiekierski et al., 2012).

Ni-Ti alloy, commonly referred to as Nitinol, is frequently used in orthopedic clinics in the form of implants such as bone plates, patellar claws, and Ni-Ti alloy sutures. Because of its good shape memory effect and characteristics that are close to but still different from human cortical bone (Wang et al., 2022). Nevertheless, orthopedic implants still face challenges from postoperative complications like swelling, pain, and the potential for bacterial infection. Furthermore, the presence of cracks in the implant material can result in fatigue fractures, leading to implant loosening or failure (Wang et al., 2022). This often necessitates expensive secondary revision surgery and consumes valuable time and resources. To address these issues, researchers have begun to explore surface modifications of Ni-Ti implants. Modifying fundamental characteristics of biomaterials, like flexibility and surface composition, enables the provision of biological instructions (Yuan et al., 2010). These modifications involve altering the structure of the metal surface, including pore size, pore design, surface roughness, surface hydrophilicity, and hydrophobicity (Bansiddhi et al., 2008). Additionally, various coating techniques have been considered, such as electroplating to increase the thickness of TiO2, localized drug delivery of antibiotics, and vapor deposition of hydroxyapatite.

## 2 Ni-Ti alloy has the following basic properties and phase transformation behavior

**FIGURE 1:**
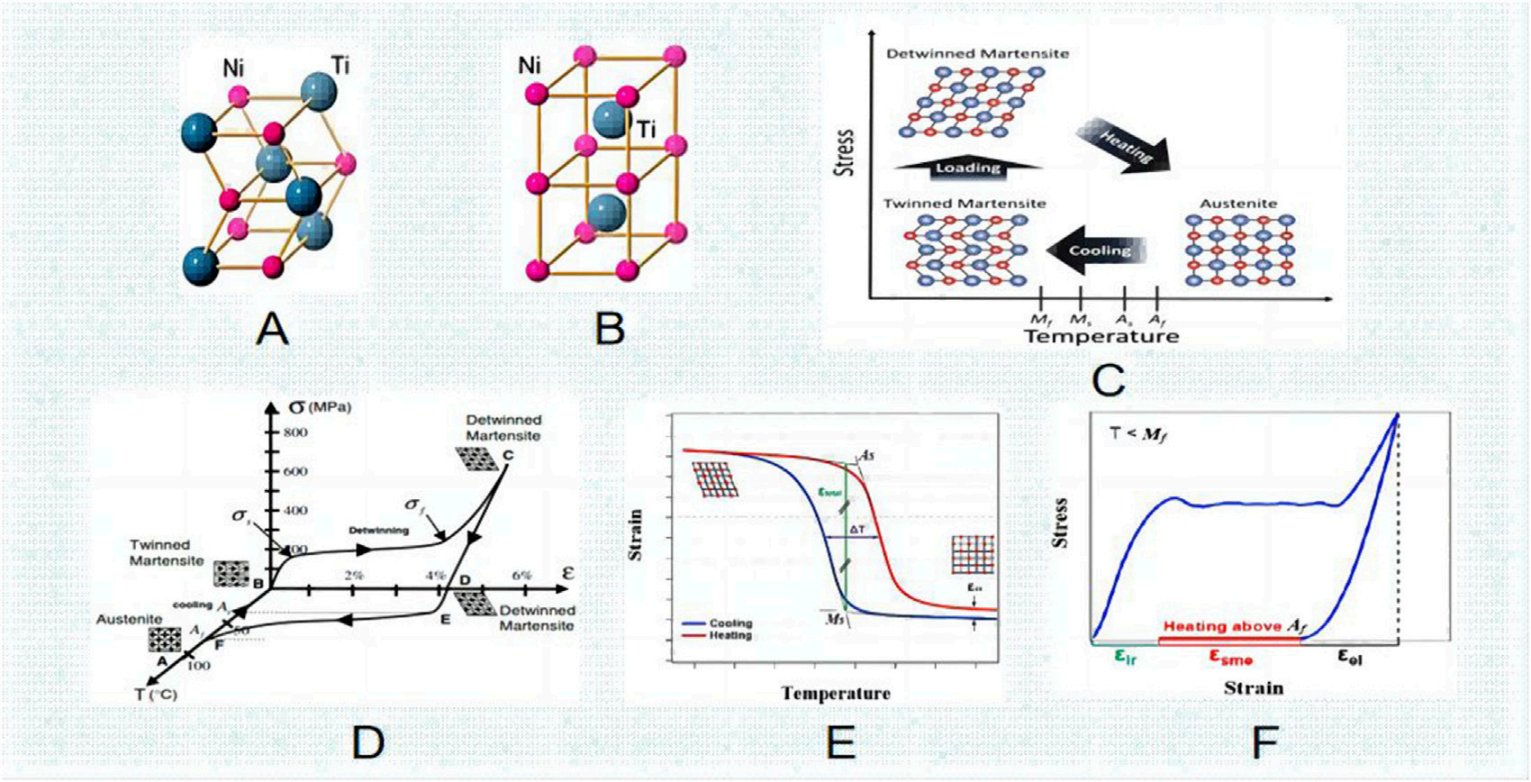
Niti Crystal structure **(A)** B190 martensite. **(B)** B2 austenite. **(C)** Crystal phase transformation diagram. **(D)** pressure and temperature phase transformation diagram. **(E)** temperature and tension phase transformation diagram. **(F)** pressure and tension phase transformation law (Khoo et al., 2018).

Ni-Ti alloy is a composition of nickel and titanium. The crystal structure of it exhibits two separate stages - the austenite phase and the martensite phase. These phases are susceptible to transition as a result of variations in mechanical pressure and temperature. The following is the phase change process that occurs when the Ni-Ti alloy cools: R phase, martensite phase, and parent phase (austenite phase). The term “austenite phase” describes a condition that is hard and has a cubic crystal structure when it occurs at a temperature higher than As, which is the beginning temperature of austenite. The austenite phase’s structure is rather constant (Figure 1B). Conversely, the term “martensite phase” describes a condition that is either initiated by an external force or occurs at a temperature that is comparatively lower than Mf, the temperature at which martensite ends. It is readily bendable, repetitious, unstable, and has a hexagonal crystal structure (Figure 1A). The Ni-Ti SMA has the ability to transition from the thermoelastic martensite phase to the austenite parent phase when subjected to heat (Saedi et al., 2018). Moreover, the plastic deformation of Ni-Ti shape memory alloy in the martensite state at low temperatures can be reversed to its original shape in the parent phase when heated, thus exhibiting superelasticity (Bansiddhi et al., 2008).

**FIGURE 2:**
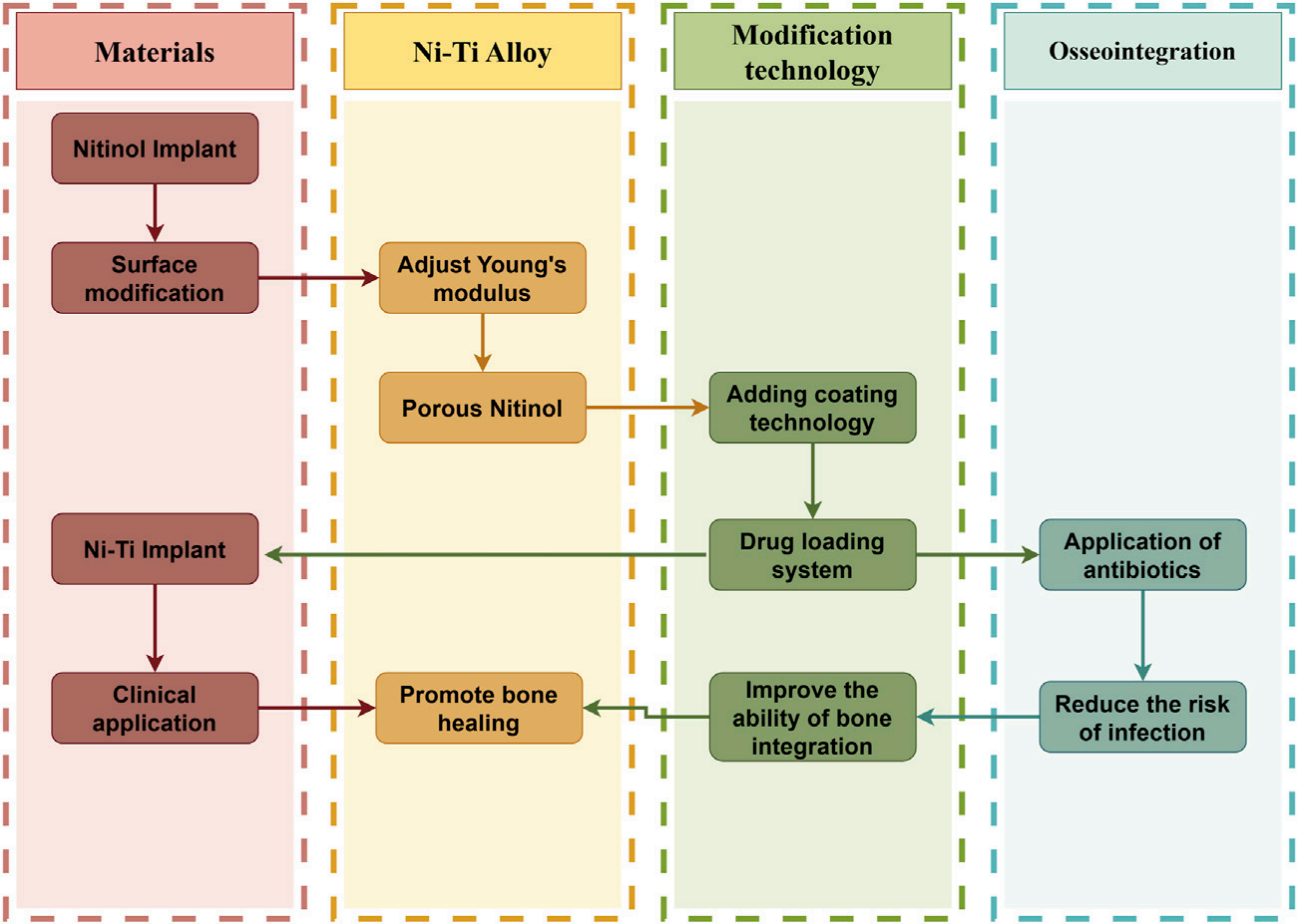
Logical schematic diagram of the effect of surface modification of Ni-Ti implant on osseointegration of implant surface (by Figdarw).

The martensite turns into austenite and reverts to its intended shape when the temperature of the Nitinol is raised above the transition point. Consequently, the “thermal memory effect” is another name for this shape memory phenomenon (Wang et al., 2022). By utilizing the alloy composition and thermomechanical treatment, one can determine the transformation temperature, as well as the As, Mf, Ms, and Af temperatures associated with austenite and martensite. They may vary from minus 150°C–200°C (about -20°C–120°C for commercially available alloys), enabling the creation of unique shape memory effects (Bansiddhi et al., 2008). Following insertion, the implant’s unidirectional shape memory effect (SME) is activated by induction heating (Figure 2). With an increase in temperature up to as, the outer sheet starts to revert to its initial (linear) form. After the implant cools to body temperature, this form is maintained because of our one-way effect. As and Af are calibrated to be much higher than body temperature but still low enough to prevent tissue injury. In order to maximize fracture healing, implant conversion can be initiated at the appropriate moment.

### 2.1 Young’s modulus of elasticity

The elasticity of metal alloys used in orthopedic implants is a significant feature, indicating their capacity to withstand deformation and revert to their initial form. Young’s modulus of elasticity is a physical measure that can quantify this property. The elastic modulus of human cortical bone ranges from 10 to 30 GPa (Jian et al., 2015). Ideally, when using an alloy for implantation, it is important for the Young’s modulus of the alloy to closely resemble that of bone. This similarity helps in avoiding the phenomenon called ‘stress shielding effect’. This term describes the decrease in bone mineral density around the implant caused by the majority of mechanical loads being absorbed by the device. Normally, the bone adapts and reshapes itself in response to applied load. But as a result of decreased stress over time, bone atrophy can happen, which might eventually lead to implant failure and loosening. In this application, alloys based on cobalt, titanium, and stainless steel are frequently utilized. The stress shielding effect of nickel-titanium alloy (NiTinol) is the smallest. In comparison to stainless steel, cobalt-chromium (Co-Cr) alloy and other metal biomaterials, Nitinol has the lowest elastic modulus, although it is still higher than that of bone tissue. Nitinol’s Young’s modulus ranges from 40 to 75 GPa, making it the ideal selection for biomedical purposes (Zhang et al., 2017).

### 2.2 Relationship between pore size, porosity, and osseointegration of Ni-Ti alloy

Porous metals have been identified as suitable candidates for the repair or replacement of damaged bones due to their adjustable hardness and porosity (Wang et al., 2016). The properties of bone can be matched by altering the porosity level, which in turn adjusts the Young’s modulus of Ni-Ti alloy. Prior research has indicated that the permeable arrangement of metals can efficiently decrease the Young’s modulus of the alloy. The pores of the alloy impact the Young’s modulus of the material and enhance the osseointegration of the implant by promoting the growth of internal bone tissue (Saedi et al., 2018). By increasing the porosity in the alloy, the strength and elastic modulus decrease in a linear manner, providing a theoretical foundation for the creation of metal implants that possess an elastic modulus comparable to that of human bones. Jane et al.’s (Khoo et al., 2018) approach of creating pore diameters in powder metallurgy was utilized to create five different types of Ni-Ti alloy samples. Powdered ammonium bicarbonate of high purity was employed as a porosity regulator and temporary retention agent. After being reimplanted, it promotes the development of adjacent blood vessels and the restoration of bone tissue by reducing the Young’s modulus of the metallic substance. When compared to pure porous titanium alloy, the superelasticity of porous Ni-Ti alloy, which resembles natural bone, results in superior bone integration and bone conductivity.

TGF-b1, or transforming growth factor β-1, is a member of the TGF-b superfamily, which is quite broad in terms of cytocompatibility. These growth factors are crucial for both adult bone remodeling and embryonic development. It is believed that osteoblasts and osteoclasts create the signaling chemical TGF-b. Anita’s research revealed, for the initial instance, the impact of Ni-Ti shape memory metal on the production of osteoblast cytokines. It specifically highlighted how the rough surface of NiTi can stimulate the expression of TGF-b1 in ROS-17/2.8 cells. The enhanced effectiveness of porous Ni-Ti alloy compared to Ni-Ti alloy can be ascribed to the greater contact surface area between the porous structure and the bone tissue, facilitating enhanced cellular attachment to the implant and the surrounding damaged bone tissue. Overall, a larger porosity generally results in better friction resistance due to the reduced contact area, and higher porosity impacts wear resistance in porous Ni-Ti alloys (Wang et al., 2016).

But is that really the case? According to Table 1, an increase in the porosity of the Ni-Ti alloy leads to a significant decrease in the Young’s modulus. At porosities of 45% and 58%, the Young’s modulus exhibits similarity to that of human bone. Nevertheless, the alloy’s increased brittleness is evident as the critical stress for plastic deformation diminishes. This implies that the service life of the alloy in the body becomes shorter. In another study, scaffolds require 50%–80% open porosity for vascularization and bone growth (Salgado et al., 2004). To improve protein absorption, porous implants utilize small pores that are less than 20 μm, while large pores exceeding 250 μm are employed for bone formation and nutrient transportation, thereby ensuring the best possible bio integration (Biggemann et al., 2018).

**TABLE 1:** Variation of elastic modulus with porosity of Ni-Ti alloy at body temperature (Litak et al., 2022).

Porosity (%)	0	32	45	58
Young’s modulus (GPa)	47	18	13	9
Critical stress for plastic deformation (MPa)	1224	503	398	300

Nevertheless, the fracture strength of porous Ni-Ti alloy does not match that of natural bone, indicating an area of improvement for porous Ni-Ti alloy. Studies by Wolfarth and Ducheyne indicate that the maximum fatigue strength of porous/solid mixed materials is only 1/3 of that of full solid samples. Additionally, comparing porous and bulk titanium alloys reveals that the fatigue strength of full porous samples decreases accordingly, and porous samples often exhibit a high corrosion rateTable. The reason for this can be attributed to both the larger surface area and the prevention of passivation layer formation. However, in the clinical practice of orthopedics, Ni-Ti alloy with porous structure is not the most popular choice in most cases, because of its low mechanical properties, especially fatigue properties. Bassani et al. (2014) Self-propagating high temperature synthesis (SHS) was used to create porous NiTi, Ti_2_Ni, and TiNi_3_ shape memory alloys. The findings indicated that the compatibility with living organisms of Ti_2_Ni was similar to or possibly superior to that of NiTi. In contrast, TiNi_3_ exhibited a high rate of nickel ion release, resulting in a lower outcome than anticipated.

SMA based on NiTi has a porous structure and is a special and compatible environment for cell hatching. It has been proved to be effective in the field of tissue bioengineering and artificial tissue development. Angiogenesis and invasion of surrounding bone tissue cells are two processes of bone repair in porous implants (Biggemann et al., 2018). The ability of porous metals to adapt to bone tissue development is another benefit of accelerating the process of osseointegration (Kapanen et al., 2002a). In an experiment conducted by Liu et al., porous nickel, porous titanium, dense nickel-titanium and dense titanium were implanted into a 5 mm diameter hole in the distal femur/tibia of rabbits for 15 weeks. The results showed that the porous SMA based on TiNi could differentiate into osteoblasts and chondrocytes and could be used as incubators. In the experiment conducted by Rhalmi et al. (1999), porous metal was implanted into the tibia and muscle back of rabbits. The findings indicated that bone remodeling took place in the bone fracture, with a notable presence of active osteoclasts and osteoblasts. The muscle tissue exhibited a thin fibrous capsule around the compact implant, with fibers permeating into the implant holes. This result is confirmed with the experimental results of Jane et al.

### 2.3 Promoting effect of hydrophobicity and hydrophilicity on osseointegration of Ni-Ti alloy

It is well-known that any foreign substance coming into contact with blood can cause complex interactions, including protein adhesion, platelet activation, and aggregation, leading to thrombosis (Scheraga, 2004; Anitua et al., 2013a; Anitua et al., 2021). Long-term anticoagulant usage is advised to reduce thrombosis. These anticoagulants, however, are only beneficial in the first phases and can lead to bleeding, clotting problems, and other illnesses in the future. Besides protein adsorption, the cell adhesion and diffusion also depend on the wettability of the material surface, as stated in reference (Witkowska et al., 2021). Here are some possible effects of the surface physical and chemical properties of this Ni-Ti alloy on cell adhesion: One of the advantages of non-wetting surface is that it reduces the amount of protein adsorbed on the surface and the adhesion strength of molecules. Another benefit is that the structural alteration of the adsorbed protein could be triggered by interaction with various molecular locations on the material’s surface. Conformational changes could potentially lead to the differential expression of ligands that interact with cell receptors.

**FIGURE 3:**
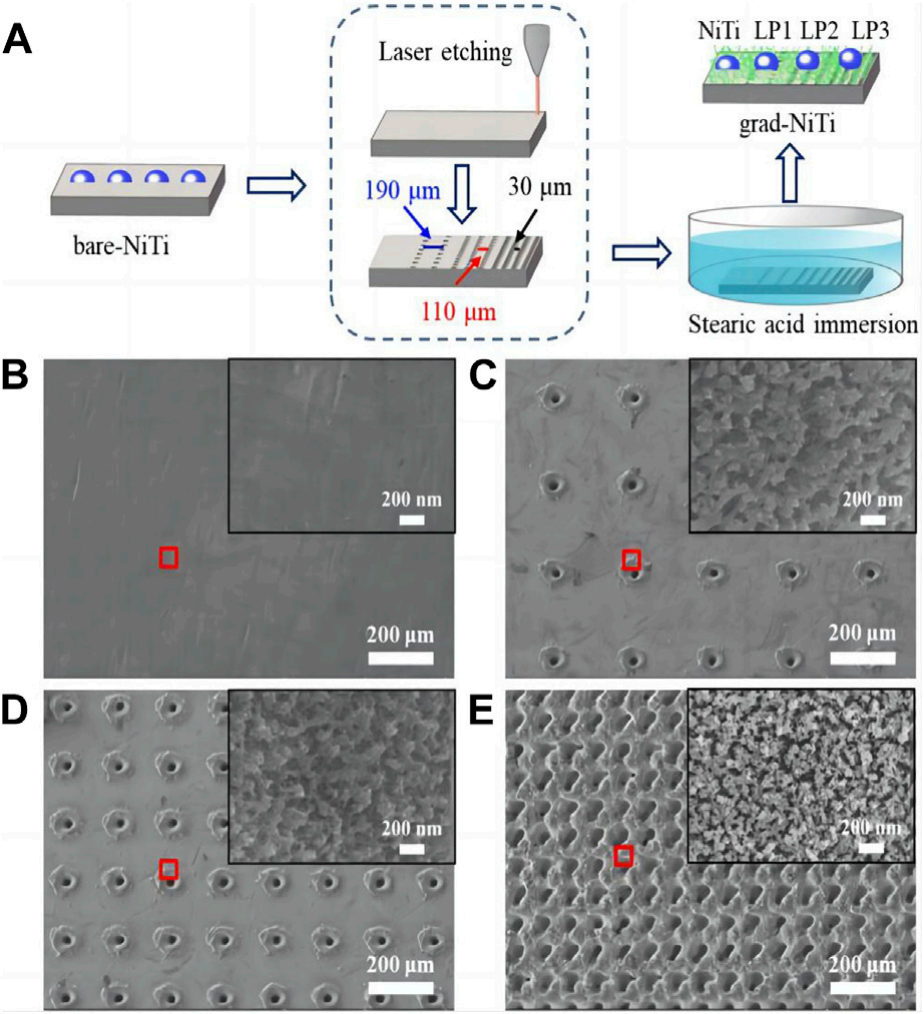
**(A)** A schematic representation of the wettability gradient surface production method. The enlarged microscopy image in the red box of **(B–E)** SEM images of NiTi **(B)** LP1 **(C)** LP2 **(D)** LP3 **(E)** the illustration shows an enlarged scanning electron microscope image of the area in the red box of b-e (Zhang et al., 2020).

Zhang et al. used surface stearic acid self-assembly and laser micromachining to create water-soluble gradient surfaces on NiTi model substrates. The findings show that the water absorption gradient surface has better anti-adhesion than exposed NiTi, which promotes blood compatibility and therefore shows a reduced hemolysis rate. In addition, compared to exposed Ni-Ti, the water-holding capacity gradient surface shows reduced flow resistance. These results suggest that thrombosis can be decreased by using the created wettability gradient surface (Zhang et al., 2020). The specific process is as follows (Figure 3):

The Ni-Ti alloy rectangular sheet is mechanically ground with silicon carbide paper. Then, ultrasonic cleaning is carried out using deionized water and absolute ethanol. Utilizing ultrashort pulse laser etching technology, three different micro-sized pores are created on various surfaces of the NiTi alloy. Afterward, the three types of Ni-Ti alloy samples with varying pore sizes are immersed in a .02 m stearic acid ethanol solution at room temperature. After soaking for 1 h, the specimens are taken out and subjected to drying in an oven at a temperature of 120°C for a duration of 20 min in order to achieve a surface with a gradient of wettability in the NiTi alloy.

In the experiments conducted by Lai et al. (2010), a gradient ranging from highly water-repellent to moderately water-repellent was achieved using the microstructure scattering technique, employing decyl trichlorosilane (DTS) as a saturate and self-assembled-monolaye (SAM). Additionally, the transition from hydrophobic to hydrophobic was created via vapor diffusion SAM. With this method, a saturated surface area matrix and a wettability gradient are created on the wafer surface below the fluid surface by placing the textured wafer vertically in a bottle holding decyl trichlorosilane solution. The smooth silicon wafer was uncovered by CI_3_Si (CH_2_) 9CH_3_ vapor, leading to the required variation in surface free energy on the wafer’s surface. This change affected the angle at which liquid and solid meet and produced a surface with varying levels of water repellency, causing water droplets to move against gravity from the hydrophobic section to the hydrophobic end (Irianov et al., 2014). Nevertheless, this method’s drawback is that it is challenging to pinpoint the gradient’s location precisely (Wang et al., 2016). In the experiments conducted by Malvadkar et al. (2010), it is mentioned that a surface with a higher solid-liquid contact angle may also exhibit viscous wettability. For example, the equilibrium contact angle on a butterfly’s wing has been measured to be more than 150°, but water droplets may still adhere to the wing even when it is in a vertical position. During the experiment carried out by Tateshima et al., NiTi tablets were subjected to 48 h of exposure to ultraviolet radiation, leading to the conversion of the NiTi surface into a state that attracts water. The modification led to an augmentation in the adsorption of albumin, resulting in a substantial improvement in the migration, attachment, proliferation, and metabolic function of endothelial cells. It has been demonstrated that the biocompatibility of NiTi surfaces treated with ultraviolet radiation has been improved with vascular endothelial cells, which has clinical implications for reducing *in situ* thrombosis formation and the need for antiplatelet drugs (Satoshi et al., 2018).

Nozaki et al. (2015) used femtosecond laser technology to modify the surface of endovascular stents and prepared hierarchical periodic micro/nanostructures on Ni-Ti alloy, which is an equiatomic Ni-Ti alloy. Two types of surfaces were created by varying the laser flux: one with micro/nanostructures and the other with periodic nanostructures. According to the assessment of the water contact angle, the micron/nano surface is hydrophilic whereas the nano-surface is hydrophilic. On the Ni-Ti alloy substrate, layered periodical arrays with nanoscale and/or microstructure might be produced thanks to this surface modification process. The study examined how the surface properties and structure influence the interaction between the surface and endothelial cells or platelets. In order to produce a solidified drum-crater-pore-particle composites structure, Feng et al. the surface of Ni-Ti alloy was altered by implementing electrically discharged machining (EDM) with the assistance of a magnetic field. The Ni-Ti alloy surface’s hydrophobicity was enhanced by this composite structure, which is made up of a solidifying bulge, crater, pore, and particle. It also decreased platelet adhesion and activation on the material surface. Moreover, the hydrophobic surface extended the duration of the drug’s activity by facilitating the drug’s gradual release. HAO et al. (Pipattanachat et al., 2021) conducted a study where they fabricated porous structures with thermosensitive poly (N-isopropylacrylamide) (PNIPAM) hydrogels supported by nickel-titanium alloy (NiTi) substrates. The majority of bacteria are unable to attach to the surface of the sample at temperatures of either 25°C or 37°C.Furthermore, the PNIPAM hydrogel possesses a pliable and biocompatible nature, thereby enabling enhanced cell adhesion and proliferation compared to the exposed NiTi alloy. Before being implanted, the NiTi sample with a porous structure and PNIPAM hydrogel that is sensitive to temperature exhibits antibacterial characteristics. After implantation, it serves a dual purpose of preventing bacterial adhesion and enhancing cell adhesion and growth, indicating promising potential in biomedical areas like orthopedic implantation.

## 3 Enhancing impact of surface alteration of Ni-Ti implant on osseointegration

**FIGURE 4:**
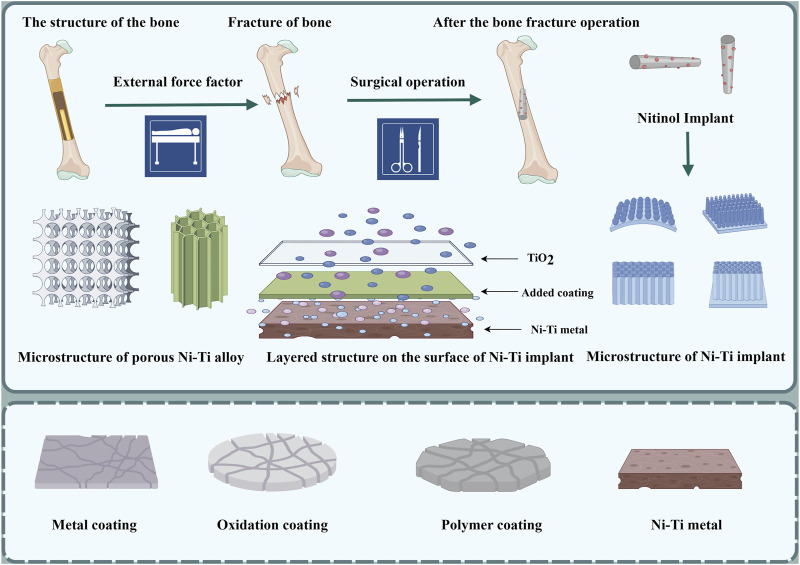
Process of implantation of Ni-Ti implant; schematic diagram of Ni-Ti implant coating and its microstructure; layered structure of Ni-Ti implant: the lowest layer is nickel-titanium metal; the middle layer is the coating of other elements added, and the top layer is titanium dioxide thin film (by Figdarw).

It is well known that the roughness of the material surface and the coating added will have an impact on osseointegration (Iijima et al., 2012; Mezeg and Primožič, 2017), and Ni-Ti alloy is no exception. Scholars have begun to realize this problem, and what arises at the historic moment is the modification of the material surface and the design of adding coating (Mezeg and Primožič, 2017) (Figure 4). The slow deterioration of materials brought on by their interactions with the outside world is referred to as corrosion. Numerous ions, including sodium, magnesium, potassium, calcium, chloride, phosphate, and bicarbonate, are present in this environment and may contribute directly to corrosion. When Ni-Ti alloys are implanted *in vivo*, the body starts to corrode and releases metal ions, which can lead to biological problems like toxicity, hypersensitivity, and cancer (Li et al., 2010). This section presents various techniques for altering the surface of Ni-Ti alloy and explores the impact of applying coatings on osseointegration (Lu et al., 2021). The coating on the nickel-titanium implant and the sorted table are described below (Table 2).

**TABLE 2:** Recent advancements in the application of osteogenic coatings on Ni-Ti implants.

Categories	Main methods or substances	Species of cell	Osseointegration function	References
Metal element oxidation coating	ZnO nanocoating	*Staphylococcus aureus*, *Staphylococcus* pyogenes and *Escherichia coli*	antibacterial activity↑	Hammad et al. (2020)
			frictional forces↓	
	Titanium nitride (TiN) coating		corrosion resistance↑	Sugisawa et al. (2018)
			frictional forces↓	
	Nanocrystalline Ti(OxNy)_2_ (rutile) surface layer	Coliform bacteria	corrosion resistance↑	Tarnowski et al. (2021)
	RFCVD process	platelet	wettability and surface free energy↑platelet adhesion↑Ni^2+^ release↓	Chlanda et al. (2018)
	TiO_2_ and a-C: N:H+TiO_2_ composite layers			
	Ag-SiO_2_ nano coating		Biological activity↑	Dulski et al. (2020)
	Ni_43.5_Ti_45.5_W_11_ (at%) NiTiW shape memory alloy	Platelet-rich plasma (PRP), Murine fibroblast cells (L929) and Human Osteosarcoma cells (MG63)	X-ray radiopacity↑corrosion resistance↑biocompatibility↑	Li et al. (2016a)
	Silver coating	L. acidophilus	antibacterial activity↑	Mhaske et al. (2015)
	Ti‒Ni‒Cu	L929 and MG63	antibacterial activity↑corrosion resistance↑biocompatibility↑	Li et al. (2016b)
		*Staphylococcus aureus*, *Escherichia coli*		
Polymer Coating	Nickel-titanium-oxygen nanopores	pre-osteoblastic cells	corrosion resistance↑, Ni2+ release↓cytocompatibility and antibacterial ability↑	Hang et al. (2018)
		*Staphylococcus aureus*		
	Rhombohedral NiTiO_3_ nanosheets	Osteoblast, penicillin and streptomycin	corrosion resistance and cytocompatibility↑Ni2+ release↓	Hang et al. (2019)
	Au at LDH/B layered double hydroxides (LDHs)	The cholangiocarcinoma cell line RBE	Anti-tumor activity↑	Wang et al. (2018)
	LDH/Butyrate	*Staphylococcus aureus* and *Escherichia coli*	Anti-tumor activity↑antibacterial activity↑	Wang et al. (2017)
		The cholangiocarcinoma cell line RBE, hepatoma carcinoma cell line SMMC-7721 and breast cancer cell line MCF-7	Ni2+ release↓	
	Ni (OH)_2_ and Ni−Ti LDH	The hepatoma carcinoma cell lines SMMC7721 and HepG2, cholangiocarcinoma cell line RBE, human intrahepatic biliary epithelial cells (HIBEpic)	Anti-tumor activity↑	Wang et al. (2015)
	Ag nanoparticles (AgNPs)/polylactic acid (PLA)/Al_2_O_3_	*Staphylococcus aureus* murine fibroblasts (L929)	thermal insulation performance↑ antibacterial activity↑biocompatibility↑	Liu et al. (2020)
Nano-scale coating	NiTi crystalline-amorphous nanocomposite (NiTi CAN)		fatigue resistance↑	Hua et al. (2021)
	ultrasonic nanocrystal surface modification (UNSM)	Millipore’s Actin Cytoskeleton	biocompatibility↑ corrosion resistance↑	Hou et al. (2018)

### 3.1 Metal element oxidation coating

The process of anodizing is a fast electrochemical oxidation technique that relies on electrode reaction to create an oxide layer on the metal surface of the anode. Two oxide layers, each measuring 1.5–10 nm in thickness, develop on titanium at ambient temperature. The oxide film that forms on the titanium surface after anodizing is thicker than the oxide film that forms on the metal surface naturally. Irrespective of the duration of the treatment, electrolytic processing has the capability to enhance the thickness of the oxide coating. Following the treatment, there is a modification in the composition of the outermost layer of oxide, resulting in a decrease in the nickel’s relative concentration in that area, without affecting the bending capabilities of Ni-Ti. According to the research of Yoneyama and Hanawa (2021), electrolysis treatment can improve the shape effect memory (superelasticity) and biocompatibility of Ni-Ti alloy by reducing the content of nickel in the surface oxide layer. After subjecting the Ni-Ti alloy to electrolysis for 30 min at 50 V in an electrolyte solution composed of glycerol, lactic acid, and deionized water, it was observed that the oxide layer thickness on the alloy’s surface increased significantly, ranging from 7 to 10 times its original size. In addition, the titanium content of the coating increases and the nickel content decreases. The absence of a nickel oxide layer on the Ni-Ti alloy surface is believed to enhance its corrosion resistance. The modifications differ depending on the electrolyte’s composition, suggesting that electrolysis can enhance the thickness of the oxide layer on the surface of Ni-Ti alloy by augmenting the titanium concentration in the alloy. This, in turn, enhances the alloy’s resistance to corrosion and its ability to retain its original shape. It is important to note that the oxide film plays a crucial role in the compatibility of implants, as stated by Hammad et al. (2020) electroplated a layer of ZnO nano-coating on a Ni-Ti orthodontic wire using electrochemical deposition. The findings indicated notable antimicrobial effects of the Ni-Ti alloy wire coated with a substantial decrease in friction against *Staphylococcus aureus*, *Staphylococcus* pyogenes, and *Escherichia coli*.

The emergence of TiN coating is also an innovative technology. The corrosion resistance of orthodontic wire is enhanced by the application of TiN coating using ion plating. Furthermore, the tensile strength of the Ni-Ti wire with TiN coating diminishes, while the TiN coating offers reduced resistance. A research investigates the impact of cathode sputtering on the microstructure and properties of the titanium dioxide surface layer on NiTi shape memory alloy produced through low temperature plasma oxidation. The findings indicate that, when compared to oxygen-based plasma, cathodic sputtering in an argon-nitrogen plasma considerably enhances the thickness of the nitrogen-doped titanium dioxide layer, thereby enhancing the surface properties of NiTi implants. Furthermore, (TiOxNy)_2_ diffusion surface layer demonstrates exceptional resistance to *E. coli* colonization (Tarnowski et al., 2021).

The structure, roughness of the surface, and resistance to corrosion of a composite layer made of nitrogen- and hydrogen-modified amorphous carbon and titanium oxide [Ti(ON)] are examined in a study by Witkowska et al. A composite layer of Ti(ON) + a-CNH was produced by coupling the RFCVD technique with plasma oxidation under glow discharge in an air environment. When compared to the unmodified sample, this composite layer, which has an amorphous carbon covering 40 nm thick, greatly increases the NiTi alloy’s resistance to corrosion Chlanda et al., the structure of TiO_2_ and a-C N H+TiO_2_ composite layers on the NiTi alloy surface was investigated by Chlanda et al. (2018). According to Dulski et al., the findings indicate that the developed nanocrystalline layer of titanium oxide (rutile) demonstrates excellent platelet adhesion and has the potential to decrease the release of Ni2+ ions. In the future, the development of coatings could be guided by the application of electrophoretic deposition to add Ag-SiO_2_ nano-coating on NiTi shape memory substrate, resulting in improved bioactivity of NiTi implants.

Li et al. (2016a) prepared three types of Ni-Ti-W alloys by introducing pure tungsten precipitation into the base phase of Ni-Ti alloy. This method utilized tungsten metal with high X-ray opacity, good cell compatibility, and corrosion resistance to enhance the overall properties of Ni-Ti alloy. According to the testing findings, the new NiTiW alloy outperformed the NiTi alloy in terms of X-ray transmission, mechanical characteristics, corrosive resistance to simulated bodily fluid, and blood compatibility. However, because of its detrimental qualities, including cytotoxicity, and metal allergy, and carcinogenicity, further research and advancements are still needed for Ni alloy wire. Arun Rameshwar Mhaske et al. (2015) conducted a study on 40 stainless steel orthodontic wires and 40 Ni-Ti orthodontic wires. The samples were separated into eight experimental groups, with each group containing 10 samples. The control group included uncoated orthodontic wires, while the experimental group consisted of coated orthodontic wires. Surface modification of the orthodontic wires was achieved through thermal vacuum evaporation of silver. Following that, a microbiological examination was performed on the orthodontic wires to assess the acidophilus resistance and antibacterial characteristics of the silver coating. The results demonstrated that the silver-coated orthodontic wires exhibited anti-adhesion to eosinophilic bacteria compared to the uncoated orthodontic wires. However, concerns regarding the cytotoxicity of silver still exist. While the silver coating on Ni-Ti orthodontic wires can resist the accumulation of *Lactobacillus*, the optimal thickness of silver coating and the maximum utilization of silver nanoparticles require further exploration. Li. et al. (2016b) conducted an experiment where they created a novel Ti-Ni-Cu shape memory alloy by incorporating copper into the Ni-Ti alloy. The findings indicated that the discharge of toxic Ni ions from the Ti-Ni-Cu alloy was considerably less compared to the TiNi biomedical alloy available in the market, and the inclusion of the Cu alloy component decreased the Ni release. Cu alloying greatly diminished the attachment and growth of bacteria in relation to its antibacterial characteristics.

### 3.2 Polymer coating

Hang et al. the technique of hydrothermal synthesis of hexagonal NiTiO_3_ microfilaments on NiTi alloy was presented. The findings indicate that subjecting Ni-Ti alloy to hydrothermal treatment enhances its ability to resist corrosion. Specifically, the therapy effectively suppressed the discharge of nickel ions, whereas extending the duration of hydrothermal exposure enhanced the release of nickel ions, attributed to the creation of nanowires possessing a large surface area. It is encouraging that the nano-scale tablets show good support for cell growth, indicating that it is possible to achieve a good tolerable release dose. Considering its good corrosion resistance, cytocompatibility and significant specific surface area, nanosheets are expected to become safe and effective drug carriers for biomedical NiTi alloys. In the previous work, Ni-Ti-O nanoparticles with different lengths were prepared on NiTi alloy with different anodizing time. According to the findings, the creation of a nano-porous framework enhances the ability of the NiTi alloy to withstand corrosion (Hang et al., 2018). On the other hand, compared with the exposed NiTi alloy, the anodized sample showed higher Ni2+ release. The results show that, contrary to electrochemical degradation, the chemical dissolution of nano-pores mainly controls the release of Ni2+.

**FIGURE 5:**
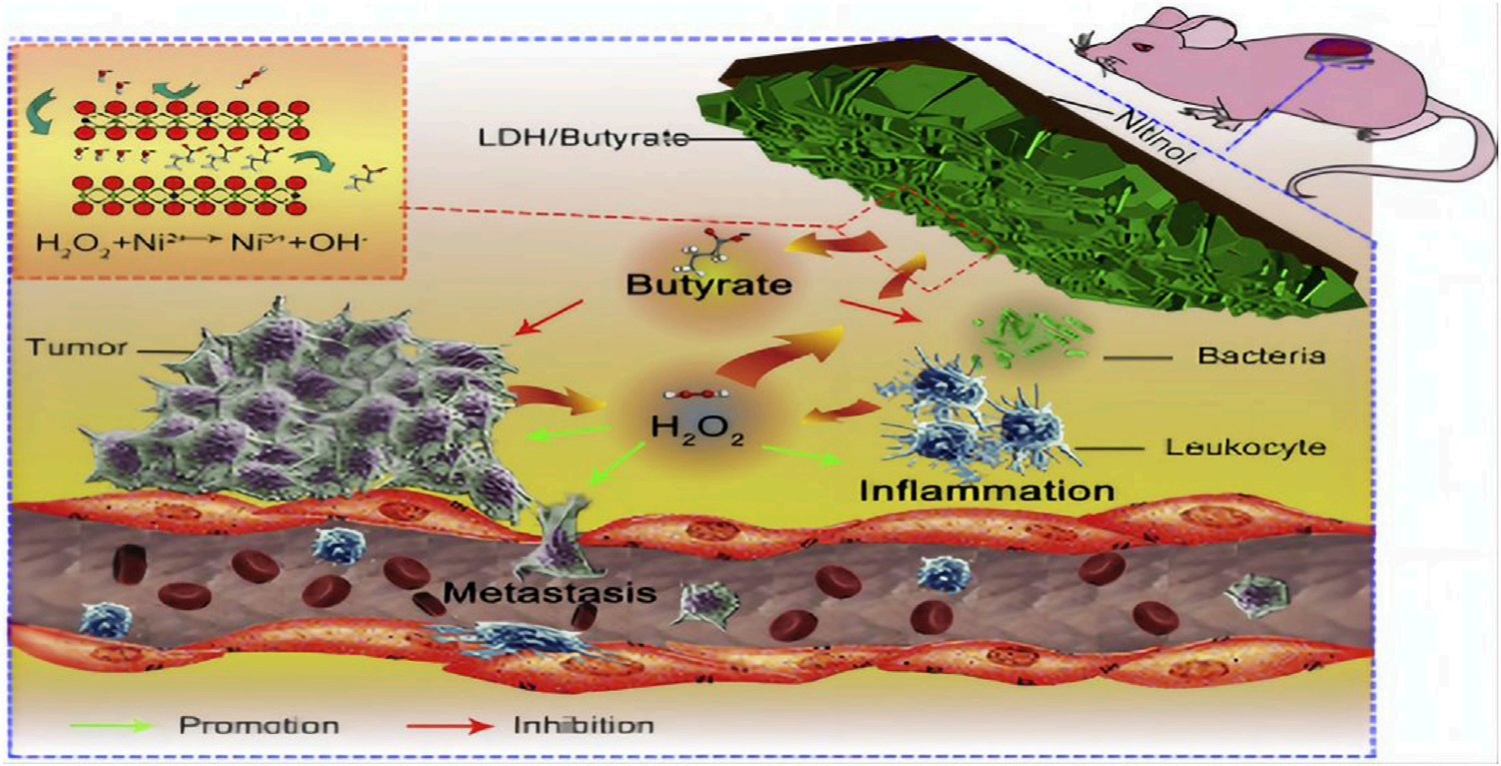
Diagram of the ability of lactate LDH/Butyrate to kill tumors and bacteria, inhibit metastasis and resist inflammation (Wang et al., 2017).

In Wang et al. a drug loading platform called Au@LDH/B, which is based on a film made of NiTi layered double hydroxide intercalated with butyrate and modified by gold nanorods, was developed on the Ni-Ti alloy surface. Research conducted in laboratory settings and living organisms demonstrates that the produced coating possesses a potent anti-cancer impact as a result of the deactivation of malignant cells caused by the inclusion of butyrate. In a separate investigation (Wang et al., 2017), a film composed of Ni-Ti layered dihydroxide (LDH/butyrate) with the incorporation of butyrate was generated on the exterior of a Ni-Ti alloy utilizing a simple hydrothermal procedure (Figure 5). The constructed membrane can use excess H_2_O_2_ generated in the tumor and infection environments to specifically suppress tumor development, metastasis, and bacterial infection. The molecule H_2_O_2_, which promotes tumors and infections, can be consumed by LDH/butyrate. Additionally, cytotoxic butyrate is injected into LDH/succinate and then exchanged. This novel coating has potential uses in the development of local drug elution systems and is anticipated to inhibit tumor growth, metastasis, and bacterial infection. In comparable trials, it was discovered that the pH-responsive barrier made up of Ni-Ti LDH and Ni (OH) 2 impeded the proliferation of malignant cells in the cell viability examination. Additionally, it was observed that the concentration of nickel and the quantity of reactive oxygen species (ROS) within cancer cells were unusually elevated, suggesting that an excessive uptake of nickel ions could potentially induce the demise of cancerous cells. The pH gradient of tumor cells could potentially be associated with the targeted suppression of cancer cells.

According to Zhao et al. after undergoing hydrothermal treatment, the NiTi alloy developed shapeless nanopores composed of Ni-Ti-O, which were then transformed into TiO_2_ and Ti_4_Ni_2_O nanopores. The findings demonstrate that the procedure may greatly increase hydrophilicity and decrease nickel ion release. In addition to encouraging the expression of endothelial function, the surface of nanoparticles stimulates macrophages, which in turn triggers a positive immunological response. To encourage healing, it upregulates M2 markers and downregulates pro-inflammatory M1 markers. Furthermore, after being cultivated in macrophage-conditioned media, endothelial cell migration, VEGF secretion, and nitric oxide (NO) generation were all improved (Zhao et al., 2021). Thus, it is anticipated that nanopores will be used as a coating for vascular stents in order to encourage re-endothelialization. Ag-doped TiO_2_, which is nanotube arrays (NTA) are a promising covering for orthopedic therapeutic implants because they simultaneously show strong biocompatibility and long-term antibacterial activity (Hang et al., 2014).

**FIGURE 6:**
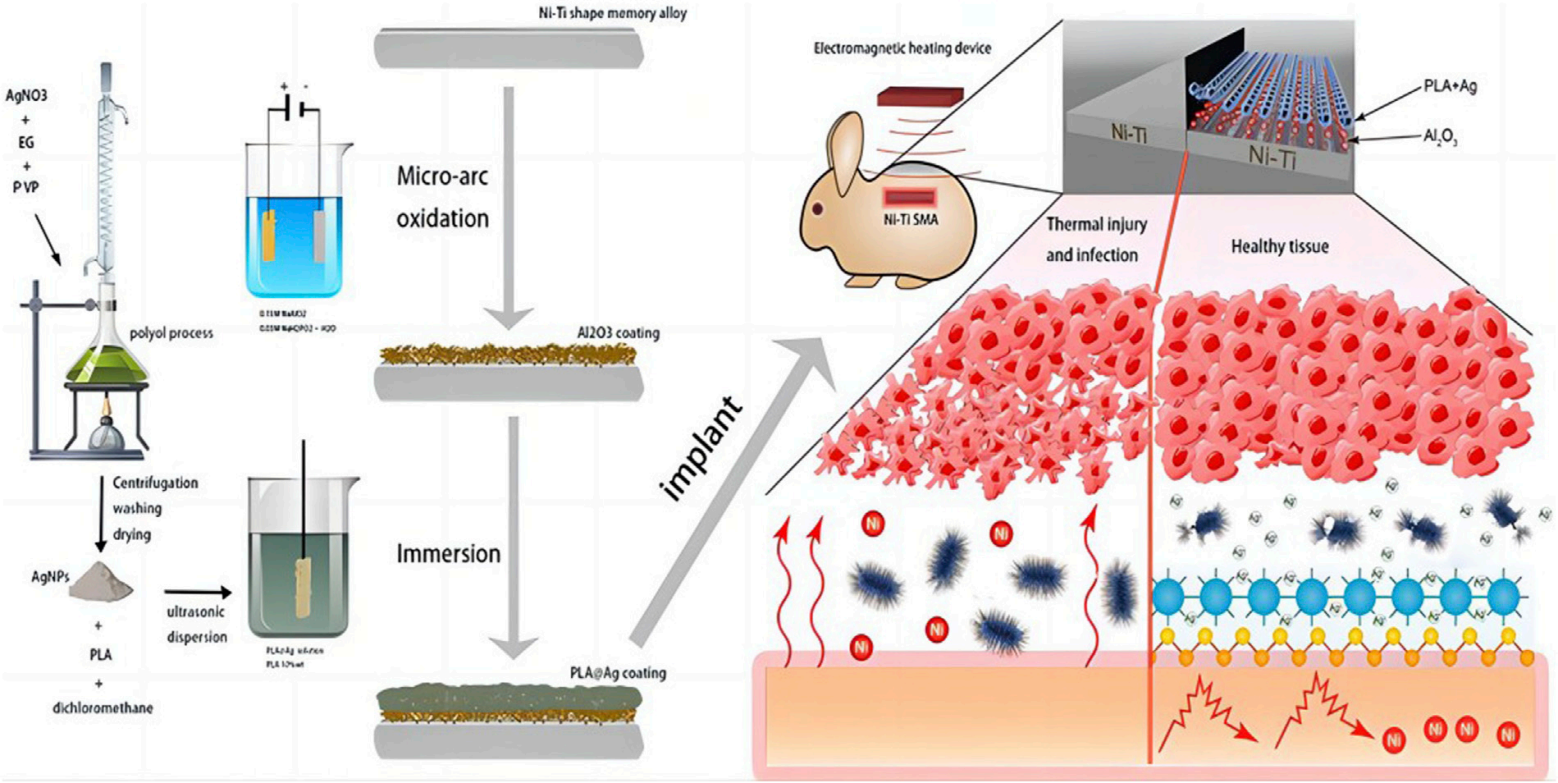
Schematic diagram of the overall Liu el study (Liu et al., 2020).

Liu et al. (2020) used multifunctional silver nanoparticles (AgNPs) with various sizes and concentrations to create an Al_2_O_3_ layer through micro-arc oxidation (MAO). They successfully prepared APA (AgNPs/polylactic acid (PLA)/Al_2_O_3_) multifunctional films by synthesizing AgNPs using silver nitrate and ethylene glycol. Due to the robust adhesion and creation of a porous surface by the Al_2_O_3_ layer, the PLA and AgNPs coatings can firmly adhere to the SMA, effectively minimizing the release of nickel ions (Figure 6. AgNPs have strong antibacterial properties and successfully stop *Staphylococcus aureus* from growing. AgNPs are firmly affixed to the PLA surface and have a high infrared reflectivity because of the poor thermal conductivity of PLA and Al_2_O_3_, which contributes to the APA coating’s superior thermal insulation capability. Nickel ion release may be isolated using a number of well-proven and practical surface modification techniques, including chemically deposited hydroxyapatite coating and micro-arc oxidation alumina coating. Preventing bacterial infections can be achieved by applying nanoscale silver or iodine coating onto the implant’s surface.

Hydroxyapatite (HA) is a mineral that primarily exists in bones and contributes to bone conduction and bone induction. One of the main clinical limitations of HA is its low *in vivo* absorption rate, along with low cleavage resistance and poor bone stimulation (Jian et al., 2015). Due to its excellent blood compatibility, carbon coating is considered a potential candidate for cardiovascular implants, such as self-expanding stents (Witkowska et al., 2021). With a nanocrystalline structure, the most thermodynamically resistant layer of titanium oxides is titanium dioxide (rutile, TiO_2_). The primary purpose of this layer is to prevent the discharge of nickel into the surrounding ecosystem, leading to a significant enhancement in the corrosion resistance of the NiTi alloy (Hua et al., 2021). The binding strength between antibiotics and the coating affects the release rate of the corresponding antibiotics in body fluids. Specifically, the chemical structure of antibiotics, particularly the number of hydroxyl and carboxyl groups, determines the acidity and basicity of the reaction solution (Stigter et al., 2004).

### 3.3 Nano-scale coating

Peng Hua et al. (2021) synthesized a sort of superelastic NiTi nanocomposites that with both crystalline and amorphous phases by using severe deformation caused by plasticity and low temperature annealing. The resulting nanocomposites exhibited a fracture resistance of 2.3 gigapascals (GPa) and a resilient deformation of 4.3%. The nanostructured Ni-Ti microcolumns underwent over 108 irreversible phase change cycles at a stress of 1.8 GPa in cyclic compression testing. The enhanced characteristics were ascribed to the reciprocal reinforcing existing between the crystalline and nano-amorphous phases. Utilizing the combined influence of the crystalline and amorphous phases at the nanoscale can effectively enhance the durability and robustness of SMA, thereby improving its resistance to fatigue and strength.

Nanoscale coating implants’ layered structure can improve the biocompatibility of the device by enhancing the interaction between cells and the implant. A layered structure on the surface was created on Ni-Ti alloy using a straightforward technique known as ultrasonic nanocrystal modification of the surface (UNSM) in a work by Hou et al. (2018) a tungsten carbide ball contacts the metal surface at an ultrasonic frequency during the UNSM process. A layered structure including embedded nano-wrinkles and micro-scale grooves is produced by the overlapping ultrasonic impacts. The findings from the cell culture experiment indicated that the samples treated with UNSM exhibited increased cell adhesion and enhanced cell proliferation. The UNSM-treated samples showed greater corrosion resistance than the untreated ones. Furthermore, there was a 22% increase in scratch hardness and a 243 Hv–296 Hv increase in surface hardness. By employing this method, the Ni-Ti alloy’s resistance to corrosion is enhanced, simultaneously enhancing its biocompatibility. UNSM has been demonstrated to be an easy and efficient way to prepare metal implant materials. Han et al. (MAINO et al., 2017) conducted a research where they utilized a dual magnetic carbon nanotube coating containing either graphene or graphene oxide on the Ni-Ti alloy substrate. The purpose was to minimize the magnetic resonance image artifact caused by the Ni-Ti alloy implant. The results demonstrated the expected effect. Iurii et al. (Irianov et al., 2014) mentioned that the reticular structure of Ni-Ti alloy can increase bone conductivity and bone induction, and Ni-Ti implants can be used as carriers for osteoblast differentiation to fill defective lamellar bone tissue. Simultaneously, the reticular framework establishes a conducive microenvironment for cells while preserving their attachment, growth, and specialization (Iriyanov Yu et al., 2013).

Laser technology has changed the surface of Ni-Ti implants, resulting in a layered structure on the nanometer/micron scale. It is worth noting that the osseointegration on the surface of Ni-Ti alloy and the process of fracture healing cannot be ignored.

## 4 Osseointegration of nickel-titanium implants’ surface

### 4.1 The process of fracture healing: staging and biomaterial surface reaction

**FIGURE 7:**
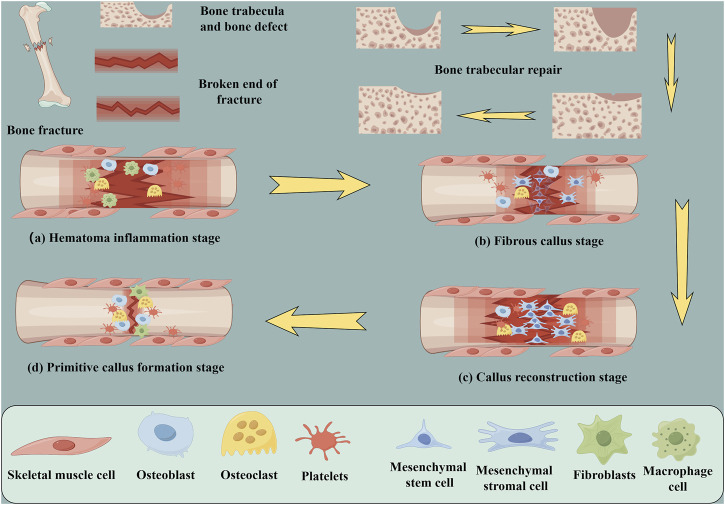
Schematic diagram of fracture repair and cellular changes; trabecular recovery process. **(A)** Hematoma inflammation stage **(B)** Fibrous callus stage **(C)** Primitive callus formation stage **(D)** Callus reconstruction stage (by Figdarw).

Although bones have the capacity for self-healing, they are unable to regenerate in the presence of extensive bone defects because there is a deficiency in the growth and differentiation platform required for bone repair-related cells (Maino et al., 2017; Cottrell et al., 2016). At present, the treatment options for large bone defects include autogenous bone transplantation (Lee et al., 2010), allogeneic bone transplantation (Larsen et al., 2011) and artificial bone graft substitute (Xu et al., 2022). In autogenous bone transplantation, there is no tissue incompatibility, and osteonecrosis after bone transplantation is a major complication (PAPE et al., 2010). However, fracture healing after implantation includes several stages, including hematoma formation, inflammation, fibrous tissue progression, endochondral ossification, callus formation and bone reconstruction (Figure 7) (Krämer et al., 2017).

The initial biological process following implant placement is the adsorption of blood proteins and the formation of blood clots on the biomaterial surface (Müller et al., 2010). The initial response of platelets to the implant materials has a direct effect on the biological recovery of Ti-Ni bone implants through the initiation of bioactive molecule release and fibrin matrix formation (Anitua et al., 2021). Growth factors and cytokines are released by activated platelets, attracting macrophages and mesenchymal stem cells (MSCs) to the surface of the implant (Huang et al., 2012; Anitua et al., 2013b; PArk et al., 2018). The presence of calcium ions enhances the process of blood clotting and aids in the creation of a prothrombin complex, which transforms prothrombin into thrombin and fibrinogen into fibrin (Wang et al., 2017; Hang et al., 2018; Zhao et al., 2021). The arrangement of the fibrin clot is intricately connected to the form and purpose of the framework on the implant surface, facilitating cell attachment, growth, and specialization.

The titanium dioxide nanotube structure refers to the formation of nano- or micron-sized tubular or porous structures on the titanium dioxide layer of commercial titanium or nickel-titanium alloy surfaces using modern industrial technology. Specifically, Titanium dioxide nanotube alloy (TNA) is considered an ideal carrier for loading bioactive elements like copper (Cu) and silver (Ag), which results in unique functions. The increased wall thickness of nanotubes provides abundant binding sites for proteins, facilitating macrophage adhesion and extension (Zhao et al., 2016). In comparison to the unmodified Ti surface, Chamberlain et al. showed that the adherence and quantity of macrophage on the nanostructure-modified surfaces were greater (Chamberlain et al., 2011).

Neacsu et al. the impact of commercial pure titanium and titanium dioxide nanotubes (with a diameter of 78 nm) on RAW 264.7 macrophages was investigated in a study (Li et al., 2010), under both conventional and inflammatory (lipopolysaccharide stimulation, or LPS) culture conditions. In comparison to the flat Ti surface, TiO_2_ nanotubes dramatically decreased macrophage inflammatory responses by means of cytokine and cytokine gene expression/protein secretion, foreign body giant cell (FBGC) activation, and nitric oxide (NO) release. This helped to mitigate LPS-induced inflammation.

### 4.2 Immune responses and implant longevity

More and more evidence shows that immunotherapy is effective in the treatment of malignant diseases, and cancer immunotherapy is widely accepted in clinic (Tan et al., 2020). In order to promote the immune response to cancer, it is always necessary to reuse immunomodulators at high doses, because most immune stimulators are unstable under physiological conditions and have poor tumor targeting ability. The emergence of drug delivery strategy is a perfect solution to this problem (Xiao et al., 2021).

As a foreign implant, it inevitably triggers immune responses, primarily through macrophage activation, which can reduce the lifespan of the implant. In the experiment of Song et al. (2018) on the treatment of ovariectomized osteoporotic mice with pulsed electromagnetic fields, it was found that the production of bone trabecular structure was related to the rapid production of osteoblasts and the inhibitory effect of osteoclasts. Furthermore, as previously stated, the production of wear particles between the implant and bone can worsen the inflammatory reaction, causing an imbalance in osteoblasts and osteoclasts, which ultimately leads to bone resorption and loosening of the implant (Brown et al., 2012). Therefore, regulating the immune response and maintaining an appropriate immune environment can facilitate improved bone union and reduce loosening.

### 4.3 The role of cell types and surface modification in the mechanism of osseointegration

The regulation of cell behavior can be influenced by characteristics of the material, including surface texture, elasticity, and chemical properties (Chen et al., 1997; Engler et al., 2006; Place et al., 2009). The osteoinductive properties of grafts depend on the type of growth factors. Several growth factors can be detected in fresh autografts (Schmidmaier et al., 2006). The effects of cell migration and differentiation are caused by various factors, including members of the transforming growth factor-b superfamily (like BMP-2 and BMP-4), angiogenic factors (such as fibroblast growth factor and vascular endothelial growth factor), platelet-derived growth factor, and insulin growth factor I.

The impaired circulatory system and bone marrow facilitate the migration of undeveloped mesenchymal stem cells (MSC) to the site of the fracture (Chen et al., 2023). In the process of hemostasis, platelets discharge transforming growth factor B (TGF-B) and platelet-derived growth factor (PDGF) to stimulate and attract undifferentiated MSC and macrophages. Initially, macrophages were called upon to eliminate waste, dead tissue, and harmful microorganisms from the site of injury (Schlundt et al., 2018). According to a recent study, mice with reduced macrophages experience impaired fracture healing. Macrophages produce fibroblast growth factor-1 (FGF-1) and fibroblast growth factor-2 (FGF-2), interleukin-1 (IL-1), and TGF- β during the inflammatory phase, which may aid in promoting angiogenesis within the bone. Additionally, mesenchymal stem cells (MSC) recruited from the exposed bone marrow, periosteum, and extraosseous stromal cells transform into fibroblasts, chondrocytes, and osteoblasts during the repair stage (Colnot, 2009).

Mesenchymal stromal cells, osteoblasts, and fibroblasts are essential for the inflammatory processes that occur in a variety of tissue types. In particular, macrophages are crucial for biocompatibility testing because of their significant involvement in inflammation and their interaction with implant materials. When inserted into biomaterials, they originate from monocytes and have a major effect on the course of wound healing (Nozaki et al., 2015; Maino et al., 2017; Pipattanachat et al., 2021). A more prominent expression of the pro-regenerative (M2) macrophages phenotype, as opposed to the pro-inflammatory (M1) phenotype, aids in tissue regeneration, including bone development surrounding the implant. This phenotype does this by reducing inflammation and promoting the osteogenic differentiation and recruitment of bone progenitor cells. Surface chemical modification with bioactive ions can further enhance the osteogenic ability of nanostructured titanium implants (Galli et al., 2014; Jiang et al., 2014). Osteoblasts exhibit a preference for rough surfaces, while fibroblasts tend to grow more on smooth surfaces (Markhoff et al., 2017).

### 4.4 Effects of nickel on cellular inflammation and angiogenesis in nickel-titanium implants

It has been discovered that ROS, or reactive oxygen species, can target and eliminate the polysaccharides on the outside of biofilms, hence having bactericidal effects on bacteria resistant to drugs (Krämer et al., 2017). Recent studies have shown that the presence of nickel ions and NiTi-NP in cells leads to the generation of ROS, resulting in the activation of chemokines and cytokines, thereby stimulating angiogenesis and inflammation (Müller et al., 2010). Furthermore, nickel triggers HIF-1α, which in turn amplifies the expression of angiogenesis genes and pathways of signaling in macrophages and endothelial cells. What sets human TLR-4 apart from mouse TLR-4 is its notable function in cell activation and inflammatory signal transduction induction.

The release of cytokines by NiTi-NP may lead to the induction of angiogenesis, which could be associated with the inflammatory process. The neovascularization of endothelial cells is largely driven by VEGF and FGF cytokines. It is important to mention that hypoxia triggers the secretion of every cytokine by M2 macrophages. Both are also expelled by endothelial cells and activated macrophages. The results shown here suggest that biomaterials and/or particles can greatly impact the processes of neovascularization and revascularization. For the first time, the findings demonstrate that nickel (as NP) may influence both processes and even promote angiogenesis *in situ* (Pipattanachat et al., 2021).

The impact of nitinol on the rat osteosarcoma cell line culture model was investigated by Kapanen et al.Unlike pure titanium, pure nickel, and stainless steel. In the 48-h culture of the rat osarcoma cell line ROS-17, the study investigated how the Ni-Ti alloy affected the rates of cell death, apoptosis, and the formation of focal contacts. The most osteoblast cell line is thought to be ROS-17/2.8, since it can express and mineralize collagen I, osteopontin, alkaline phosphatase, and osteocalcin. The experimental findings demonstrate that whereas pure titanium has a strong cell tolerance, there are less local contacts than in NiTi samples. NiTi is an osteoblast-type ROS-17 cell that is well tolerated (Kapanen et al., 2002b).

In orthopedic clinics, customized Ni-Ti implants are also required to address each patient’s unique demands and provide efficient care. Patients with severe osteomyelitis, patients with trauma-related bone abnormalities, and patients who have undergone an extensive osteectomy for bone cancers are among the patients who usually need implantation. For patients with bone malignancies, titanium implants containing anti-tumor medicines such cisplatin, adriamycin, curcumin, etc., are frequently utilized. (MA et al., 2021). Here are some Ni-Ti implants that have been used in clinic in recent years, and analyze their advantages and disadvantages in order to promote the development direction of clinical implants in the future.

## 5 Clinical application of Ni-Ti alloy in orthopedics

In clinical practice, poly (methyl methacrylate) (PMMA) cement for bones is frequently utilized as a conventional antibiotic carrier. When treating osteomyelitis, PMMA beads containing antibiotics are commonly used. However, there are disadvantages associated with the utilization of PMMA as a carrier for antibiotics (Table 3). These include the requirement of an additional surgery to eliminate the non-absorbable adhesive in patients with osteomyelitis, as well as the potential for heat-induced harm to medications due to the exothermic polymerization of the cement (Chen and Taguchi, 2019).

**TABLE 3:** The name, sample number, follow-up time and therapeutic effect of nickel-titanium implants used in clinic.

Category	Number of samples	Follow-up time	Therapeutic effect or research results	References
Four-Corner Arthrodesis Concentrator of Ti-Ni Memory Alloy	18	30 months (range, 12–48 months)	Effectively treat the wrist collapse and retain most of the wrist function	Xu et al. (2012)
Nickel–titanium (Ni–Ti) memory alloy arthrodesis concentrator	24	12 months (range, 6–24 months)	The Ni–Ti memory alloy arthrodesis concentrator is a convenient tool for scapho-trapezio-trapezoeid (STT) arthrodesis with excellent and reliable results	Author Anyonumus (2024)
Ti-Ni olecranon memory connector (OMC)	20	3.2 years (range 2–5 years)	The OMC could be an effective alternative to treat olecranon fractures	Chen et al. (2013)
Ti-Ni shape-memory sawtooth-arm embracing fixator	21	39.7 months (range, 1–78 months)	The embracing fixator is a valid alternative treatment for Vancouver type B1 or type C periprosthetic femoral fractures	Zhao et al. (2012)
Claw-like Ti-Ni SMA fixator (SMA-claw)	29	11.48 months	Ti-Ni SMA claw fixator has good bone grafting effect and can restore stress sustainably. It can replace the traditional tension band technique in the treatment of transverse patellar fracture	Hao et al. (2015)
Ti-Ni shape-memory sawtooth-arm embracing clamp (Ni-Ti SSEC)	21	48.2 months	The Ni-Ti SSEC is a simple and valid method for fixing osteotomies in treating complex femoral revision surgery	Li et al. (2016c)
Ni-Ti arched shape-memory connector (ASC) combined with partially threaded cancellous screws (PTCS)	21	65 months (range, 22–90 months)	ASC combined with PTCS can be used as an effective method for the treatment of supracondylar comminuted fracture of femur	Zhang et al. (2013)
Ti-Ni arched shape-memory connector (ASC)	108	42 months (range, 31–53 months)	The ASCs can effectively reduce the incidence of internal fixation loosening, fracture, infection and other complications	Zhao et al. (2022)
Ti-Ni shape-memory patella concentrator (TNSMPC)	54	12 months	TNSMPC combined with cannulated compression screw is an effective internal fixation method for the treatment of C2 and C3 patellar fractures without additional technical difficulty and tissue injury	Yao et al. (2020)
Ti-Ni arched shape-memory alloy connector	18	4.2 months (range 12–36 weeks)	Nickel-titanium SMA arch connector combined with autogenous bone graft can be used to treat scaphoid nonunion, which is worth popularizing	Zhou et al. (2019)

Xu and colleagues successfully utilized a Nickel Titanium Memory Alloy device called Four-Corner Arthrodesis Concentrator to treat wrist collapse, resulting in the preservation of a significant portion of wrist functionality (XU et al., 2012). A different research involved the treatment of 24 individuals who had avascular Lunate necrosis using scapho-trapezio-trapezoeid (STT) arthrodesis alongside a nitinol fusion cage. The study concluded that the nitinol memory alloy arthrodesis cage proved to be a practical instrument for achieving STT joint fusion (Author Anyonumus, 2024). Chen et al. (2013) conducted a study on 40 patients with ulnar olecranon fracture, where they randomly assigned them into two groups. Among them, 20 cases were treated with OMC internal fixation and 20 cases with locking plate. OMC outperformed the locking plate in MEP score, while there were no notable disparities in DASH score, occurrence of complications, and range of motion of the elbow joint between the two approaches. OMC is an effective method to replace Kirschner wire, tension band wire and locking plate in the treatment of olecranon fracture. Su et al. (2010) developed a swan-shaped humeral connector (SMC) and successfully treated 156 patients with humeral shaft nonunion between 1997 and 2007, achieving an impressive effective cure rate of 98.7%. No adverse reactions were observed post-operation. Additionally, Song et al. evaluated the mechanical and biological characteristics of the internal fixator known as Ni-Ti navicular arc nail (NT-SAN). The results demonstrated that NT-SAN has a long service life and possesses high strength and a strong anti-fatigue effect. The fatigue strength meets the requirements for bone healing following a scaphoid fracture.

In the article of Hao et al. (2015), it is mentioned that 29 patients with patellar fracture were treated with Ni-Ti SMA. The findings indicate that the titanium nickel shape memory alloy (SMA) claw fixator demonstrates a favorable bone grafting outcome, enables continuous stress recovery, and can serve as an alternative to the conventional tension band method for treating patellar transverse fractures. In the clinical practice of Li et al. (2016c), 21 patients with complex hip arthroplasty were treated with Ni-Ti SSEC. The average follow-up period for all patients was 48.2 months. According to the findings, the mean Harris hip score rose from 21.2 prior to the revision surgery to 83.1 during the recent examination. No implant failure or malunion occurred in all patients. This orthopedic implant is a simple and effective method to fix osteotomy for complex revision of the femur. Using a nickel-titanium arched shape memory connector (ASC) combined with partially threaded cancellous screws (PTCS), ZHANG et al. (2013) repaired 21 patients with supracondylar coronal fractures of the femur. The results showed that none of the subjects had osteonecrosis or arthritis. The combination of ASC and PTCS is a viable approach for effectively treating femoral supracondylar comminuted fractures. Nevertheless, additional future comparative investigations and biomechanical examinations are necessary to assess the lasting outcomes of utilizing these substances. Zhao et al. (2022) conducted a study involving 108 individuals who had ankle fractures accompanied by distal tibiofibular syndesmosis ligament injury. Among these patients, 72 were assigned to the SCREW group, while 36 were assigned to the ASC group. Both surgical methods, namely, ASCs and screws, were used for treatment. The findings indicated that the ASC group achieved a notably higher score in the OMAS score system compared to the SCREW group. Additionally, the ASC group exhibited a higher MOS SF-36 score compared to the SCREW group. ASC has the ability to effectively decrease the occurrence of complications like loosening of fixation, fractures, infections, and others.

**FIGURE 8:**
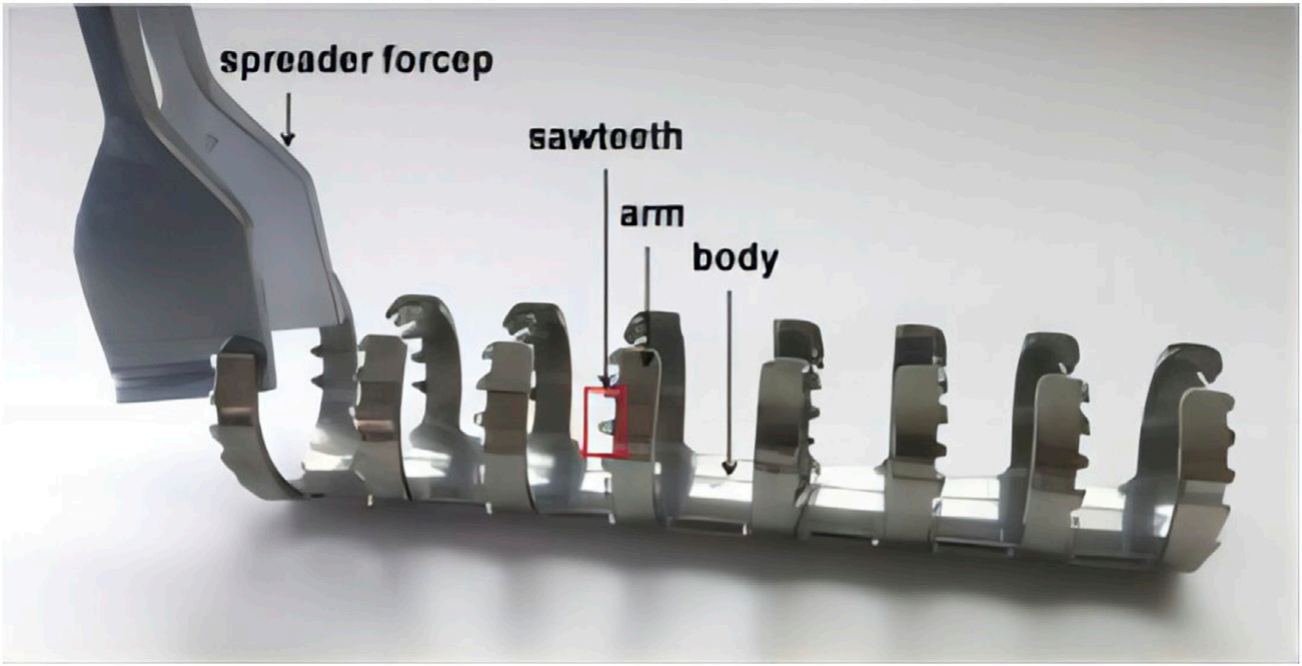
The Ni-Ti shape-memory sawtooth-arm embracing clamp (Ni-Ti SSEC) consists of the body, two arms, and a sawtooth. The arms on both sides of the osteotomy have a symmetrical shape in the axial direction. The clip’s arms can be extended using specialized expansion pliers within the temperature range of 0°C–4°C. When heated with hot brine (40°C–50°C), the arm returns to its original shape, causing the Niti SSEC to effectively seal the fracture’s end (Zhao et al., 2012).

Yao et al. (2020) conducted a clinical study where they treated 54 patients who had fractures in the patella of C2 and C3. The treatment involved open reduction and the use of a Ti-Ni shape-memory patella concentrator (TNSMPC) along with internal fixation using cannulated compression screws. The patients were then followed up for a minimum of 12 months. The combination of TNSMPC and cannulated compression screw proves to be a successful technique for internally fixing C2 and C3 patellar fractures, without any added technical complexity or harm to the surrounding tissues. The clinical data of 18 scaphoid fracture cases treated with autologous iliac arc bone connector were reviewed and analyzed by Zhou et al. (2019) all the 18 patients achieved acceptable fixation and reduction, and the average recovery time was 4.2 months. Statistical analysis revealed a notable enhancement in the recovery of wrist function, fracture healing, and simulated pain perception when compared to the pre-operation period. Moreover, based on the imaging observations of Schipper et al. (2018), additional fixation with partial threaded screws did not significantly affect the radiological fusion of Nitinol fixation. Put simply, there is no clear proof that the outer layer pattern of the arc nail can improve stability after surgery for scaphoid fractures. Periprosthetic femoral fractures can be treated using the Nickel-titanium shape-memory sawtooth-arm embracing fixator (Ni-Ti SSEF) as demonstrated in Zhao et al. (2012) study (Figure 8). The fixator provides a constant holding force to stabilize the fracture, improves bone healing and reduces osteoporosis after fixation.

The surgical treatment with Ni-Ti SSEF solves the problem of limited screw fixation space around the prosthesis and the challenge of using bone for screw fixation by using extramedullary clips. However, muscle stripping is still necessary for patients who receive NiTi SSEC, although the degree of muscle stripping is much smaller than that of bone allografts. This is because NiTi SSEC’s arm may be introduced into the area between muscle fibers, potentially promoting osteotomy repair and reducing the incidence of deep infection.

## 6 Discussion

The alloy nickel-titanium (NiTi) has become the standard material for orthopaedic and dental restorations because of its high biocompatibility and stable chemical characteristics (Gonzales et al., 2010). But there are still issues that might lead to implant removal, including as surgical loosening and peri-implant infection. The implant healing process has been shortened due to advancements in implant surface treatment technology. Two primary approaches are used to achieve bone union at the interface between the implant and bone. The first is long-distance osteogenesis, where osteocytes migrate from the bone tissue to the surface of the implant. This process is believed to accelerate bone formation and is particularly significant for immediate weight loading. The second method is contact osteogenesis, where the implant surface attracts osteoblasts and stimulates bone formation on its surface. Following the insertion of the implant into the bone, there is a simultaneous initiation of contact osteogenesis and long-range osteogenesis (Choi et al., 2017).

The nitinol implant, which is a platform for regenerating bone defects using the patient’s own cells, is intricately connected to the process of human bone marrow mesenchymal stem cells (MSCs) transforming into osteoblasts and osteoclasts. The successful regeneration of bone on the implant surface depends on the abundant production of osteoblasts and the suppression of osteoclasts (Chang et al., 2006; Ongaro et al., 2014). Contrary to previous beliefs, it has been discovered through studies that the main substance responsible for activating contact osteogenesis is bone morphogenetic protein-2 (BMP-2), which is produced by the host bone, rather than circulating bioactive proteins like platelet-derived growth factor. Jung-Yoo Choi’s experimental findings provided evidence of BMP-2 expression at the interface between bone and implant, thus reinforcing the idea of contact osteogenesis occurring on the surface of the implant (114). Certain metals like nickel, chromium, and cobalt, as well as components of bone cement such as acrylate and gentamicin, may cause intolerance reactions to implants (Giombini et al., 2007).

Klotho refers to a group of membrane proteins associated with the process of aging and diseases related to age. The expression of it is primarily observed in the kidney and it creates a compound with FGF23 receptors located on the cellular membrane (Lindberg et al., 2014; Xu and Sun, 2015). The products of the Klotho gene may have a role in regulating the occurrence of aging and diseases related to age in living organisms (Kuro-o et al., 1997). The klotho Klotho families of different species include α-Klotho, β-Klotho and γ-Klotho. According to a single study, β-Klotho hinders the insulin and Wnt signaling pathways, suppresses oxidative stress, and controls phosphatase and calcium absorption (Xu and Sun, 2015). According to recent research (Zhang et al., 2023), α-Klotho has been found to have varying associations with overall bone mineral density, bone mineral density in the thoracic region, bone mineral density in the lumbar region, and bone mineral density in the trunk. Studies involving animals have demonstrated that the expression of Klotho by osteocytes is crucial in the regulation of bone metabolism, and the targeted removal of klotho in osteoblasts leads to an elevation in bone mass. (Komaba et al., 2017).

Eczema, prolonged wound or healing of the bone, recurring effusion, discomfort, or implant loosening are some signs of implant allergy (Xiao et al., 2021). Commercial pure titanium has a special osseointegration property that makes it possible for the bone and implant tissue to join structurally and functionally without the need for the creation of a soft tissue interface, out of all the alloys that are now available. After titanium is implanted, a thin oxide layer covers it, which is followed at the micron and nanometer scale by layers of protein, cell, calcified, and bone tissue. Modifying the characteristics of an implant can be achieved through various means, including manipulating the surface’s micro and nanostructure, adjusting hydrophilicity and roughness, applying biological surface treatment, and introducing chemical components. Bacteria colonization and biofilm formation on implanted devices can lead to both short-term and long-term infections in the surrounding bones and soft tissues. Long-term high-dose antibiotic usage for these illnesses may lead to systemic and localized toxicity, drug resistance, and possible impairment of bone formation. (Liu et al., 2014).

The TC4 material is made up of Ti-6Al-4V, which is classified as a titanium alloy of the (α + β) type and possesses favorable mechanical properties overall. In comparison to Ni-Ti alloy, the Ti6Al4V implant with quercetin coating has the potential to improve the attachment and differentiation of rBMSCs, control the shift of macrophages from M1 to M2 polarization, and enhance the expression of genes related to anti-inflammatory responses and vascular functions. Furthermore, the implant infused with quercetin decreased the inflammation surrounding the implant and enhanced the formation of new bone and swift osseointegration *in vivo* (Liu et al., 2022). To encourage bone formation, a hydrogel containing bone morphogenetic protein-2 (BMP-2) was introduced into a porous Ti6 Al 4V scaffold, while osteoprotegerin (OPG) was used to prevent excessive osteoclast activity. The findings indicated that the sustained discharge of BMP-2 and OPG within the composite scaffold greatly enhanced bone formation and integration in bone imperfections (Wang et al., 2021). In an article studying the difference between sulfur oxidizing bacteria (SRB) and sulfate reducing bacteria (SRB) strains, it is mentioned that the affinity of NiTi and Ti6 Al 4V alloy to this bacteria is different. The results show that sulfate reducing bacteria strains are more adaptable to environmental changes than sulfate reducing bacteria, and the surface contamination of titanium alloy implants may pose a threat to human health (Cwalina et al., 2017).

### 6.1 The challenges encountered in Ni-Ti clinical implants and the future development direction

The advantage of porous Ni-Ti alloy in clinical application is that many implants are closest to Young’s modulus of human bone, but there is still a certain gap with the elastic modulus of human bone. However, an obvious disadvantage of porous Ni-Ti alloy is its low mechanical properties, especially fatigue properties. The future development of the porous material is worrying because of its low mechanical properties.

The toxicity and potential to cause cancer of nickel are among the primary concerns associated with NiTi surgical implants. Several research studies indicate that reduced levels of nickel can potentially promote cellular growth to a small extent. Nevertheless, when Ni reached high concentrations (approximately 15–30 mg/mL), it considerably impeded cell growth rate and induced alterations in cell morphology (Bearden and Cooke, 1980). And Ni-Ti wire can cause toxicity of human osteosarcoma cell line in corrosive solution similar to artificial saliva (Kao et al., 2007). Dissolved nickel is thought to be the cause of the detrimental effects of Ni-Ti SMA corrosion products on cultured smooth muscles cells (Chen et al., 1997). It is interesting to note that Denise’s paper mentions that Ni-Ti alloy, with up to 50% nickel content, has no cytotoxic impact on osteoblasts and fibroblasts, and that Ni-Ti SMA with a 50:50% Ni:Ti ratio has high biocompatibility under research conditions (Engler et al., 2006). Overexposure to nickel can result in cellular hypersensitivity, cytotoxicity, genotoxicity, asthma, and allergic responses, among other negative symptoms that might cause serious health issues (Xu et al., 2022). Furthermore, 4.5% of the population as a whole has nickel allergies, which might cause allergic responses to nickel-titanium alloys that are implanted (Mcmahon et al., 2012).

In terms of clinical application, the emergence of different types of Ni-Ti implants on the whole has solved the pain of fracture, but infection, secondary surgery after operation and cytotoxicity of nickel are still problems to be solved in the future.

## 7 Conclusion

The bone bonding properties of Ni-Ti implants can be improved by using different surface modification coatings such as metal oxidation coating, composite coating, nano-coating and so on. These coatings also promote the adhesion of red blood cells and platelets in the body, promote local tissue repair and the healing of fractures or bone defects. In addition, the hydrophilicity and hydrophobicity of Ni-Ti implants play an important role in osseointegration and fracture repair. In addition, compared with other metal implants, the Young’s modulus of Ni-Ti implants is closer to that of human cortical bone, although there are still some differences. This similarity helps to reduce postoperative loosening, and the addition of the coating enhances the osseointegration on the surface of the Ni-Ti implant, and finally enhances the biocompatibility of the implant *in vivo*, thus improving the success rate of the implant. So that Ni-Ti shape memory alloy implants are of great significance in orthopedic clinical application.
