# Supplementary material for: Multifunctional coatings of nickel-titanium implant toward promote osseointegration after operation of bone tumor and clinical application: a review
Source: Front Bioeng Biotechnol. 2024 Feb 20;12:1325707. doi: 10.3389/fbioe.2024.1325707 (PMC10912669; doi:10.3389/fbioe.2024.1325707)
Supplement: Supplementary file 1 [file DataSheet1.docx]

Supplementary Material

# Supplementary Figures and Tables

## Supplementary Figures


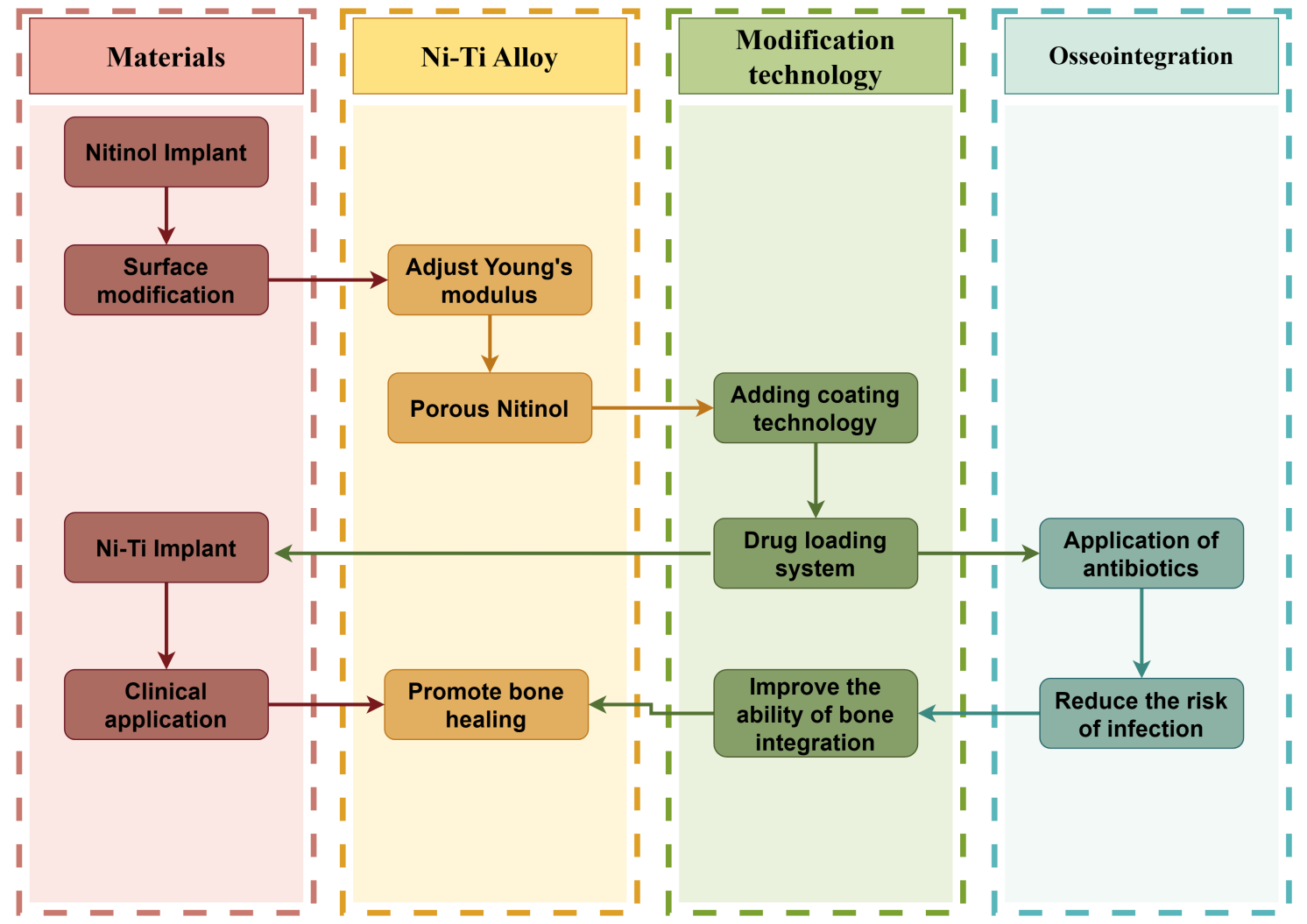


**Supplementary Figure 1**:Logical schematic diagram of the effect of surface modification of Ni-Ti implant on osseointegration of implant surface (by Figdarw).


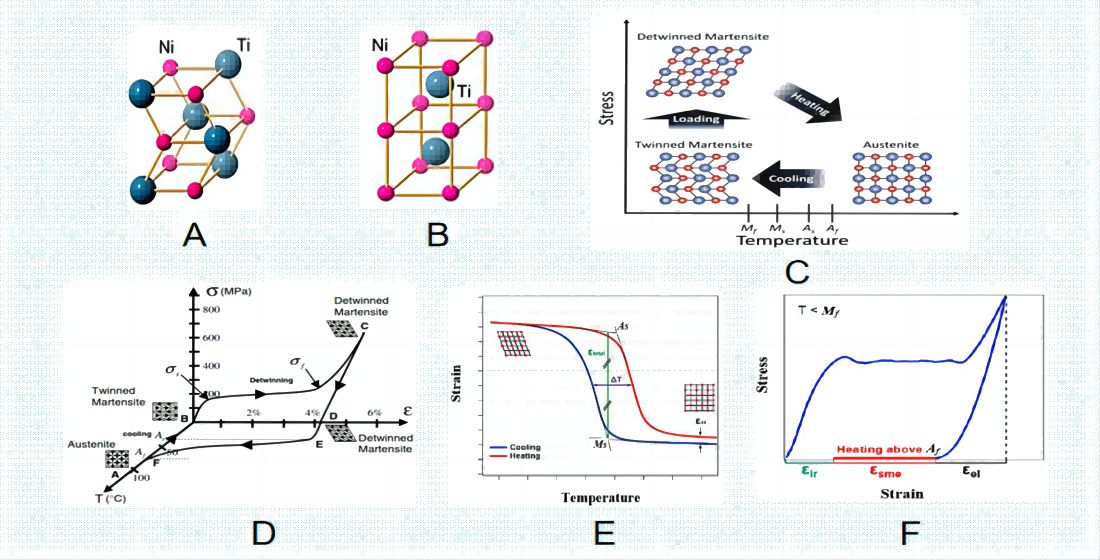


**Figure 2** NiTi Crystal structure (A) B190 martensite. (B) B2 austenite. (C) Crystal phase transformation diagram. (D) pressure and temperature phase transformation diagram. (E) temperature and tension phase transformation diagram. (F) pressure and tension phase transformation law.
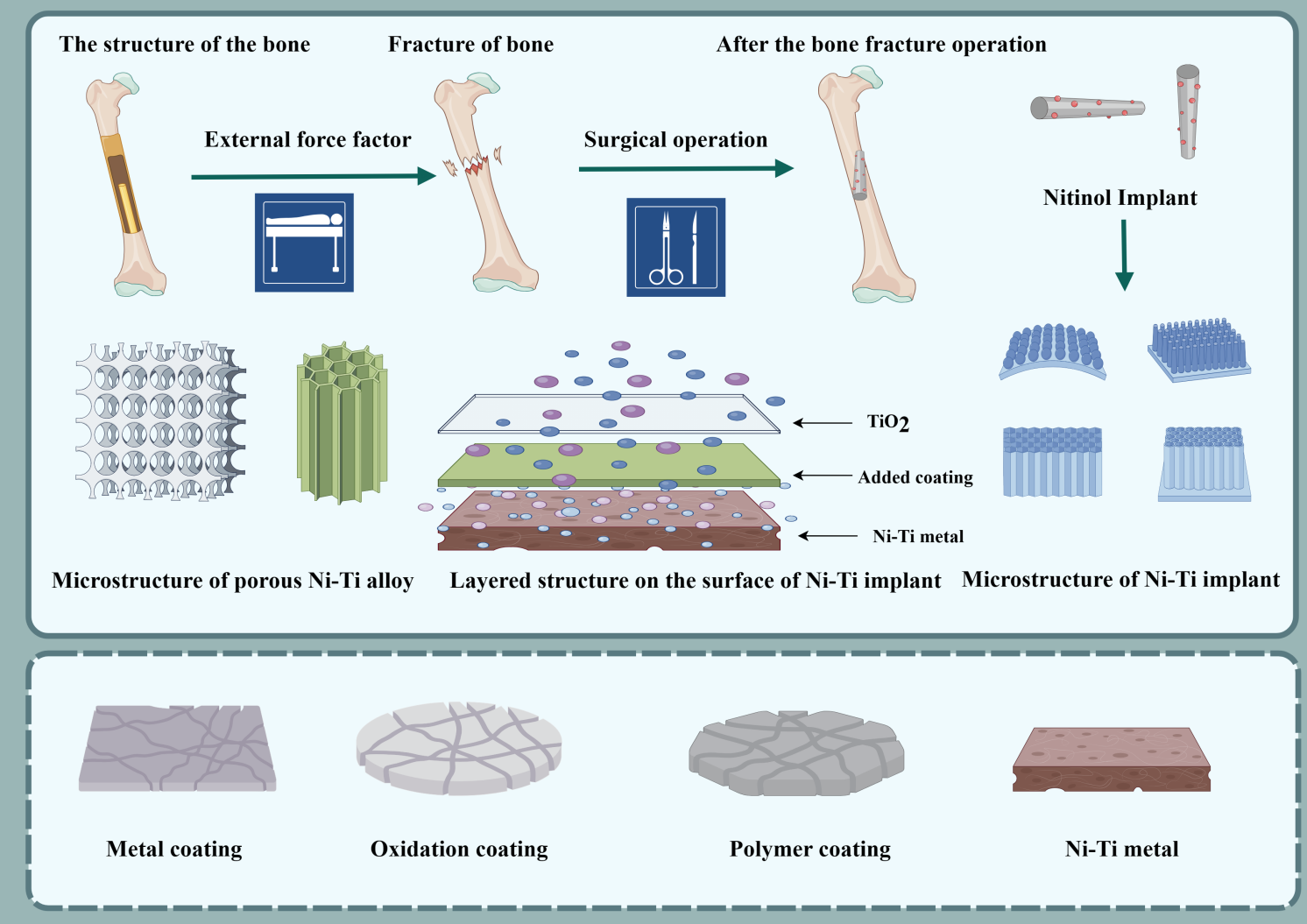


**Figure 3**. Process of implantation of Ni-Ti implant; schematic diagram of Ni-Ti implant coating and its microstructure; layered structure of Ni-Ti implant: the lowest layer is nickel-titanium metal; the middle layer is the coating of other elements added, and the top layer is titanium dioxide thin film (by Figdarw).


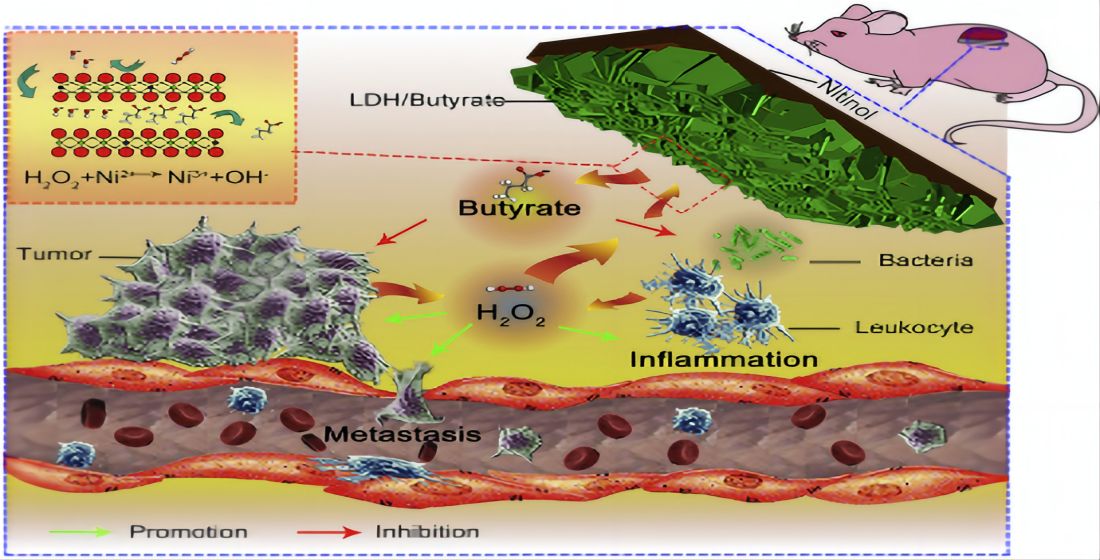


**Figure 4** Diagram of the ability of lactate LDH/Butyrate to kill tumors and bacteria, inhibit metastasis and resist inflammation.


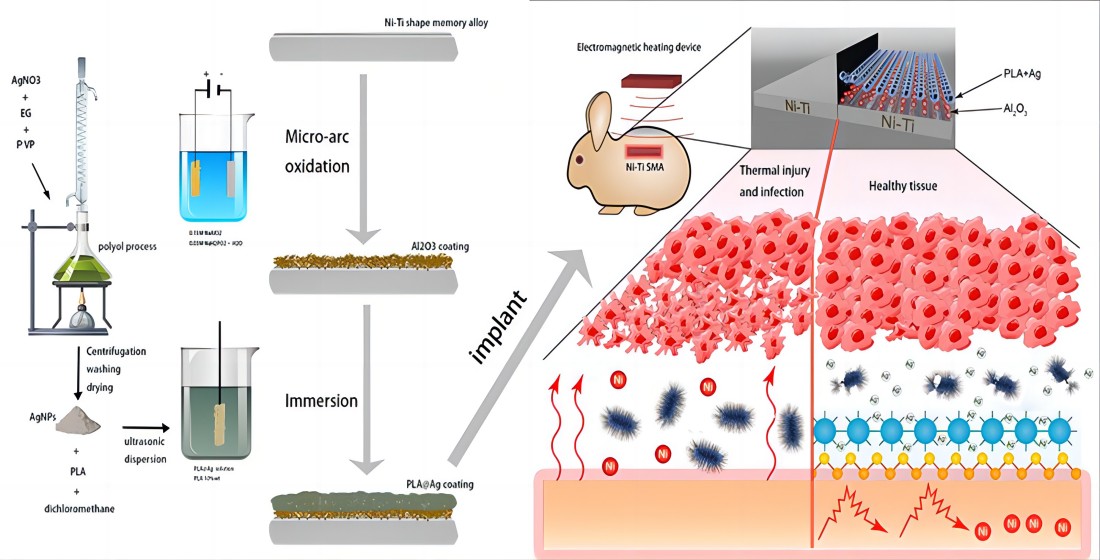


**Figure 5**. Schematic diagram of the overall Liu el study.


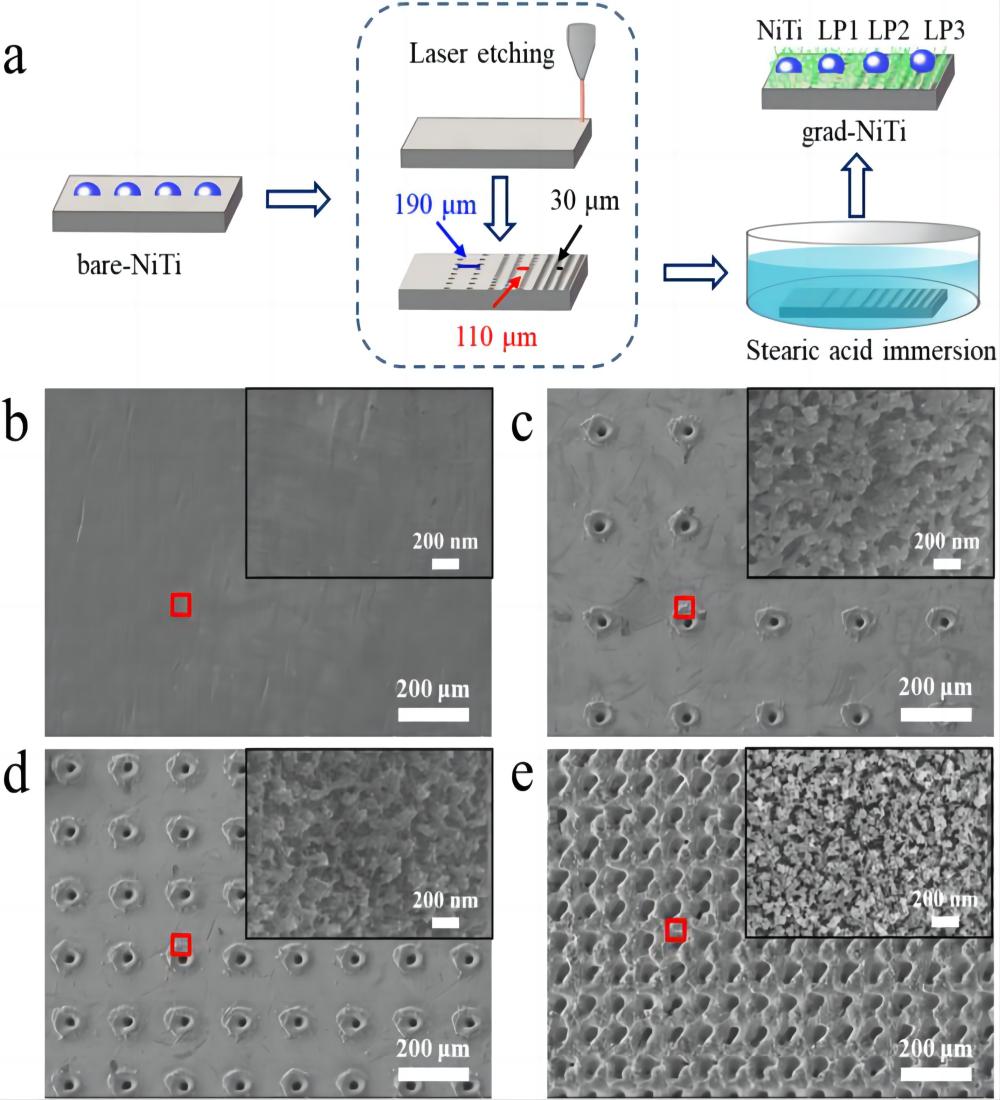


**Figure 6**. (a) Schematic illustration of the fabrication process of wettability gradient surface. (b–e) SEM images of NiTi (b) LP1 (c) LP2 (d) LP3(e) the illustration shows an enlarged scanning electron microscope image of the area in the red box of b-e.


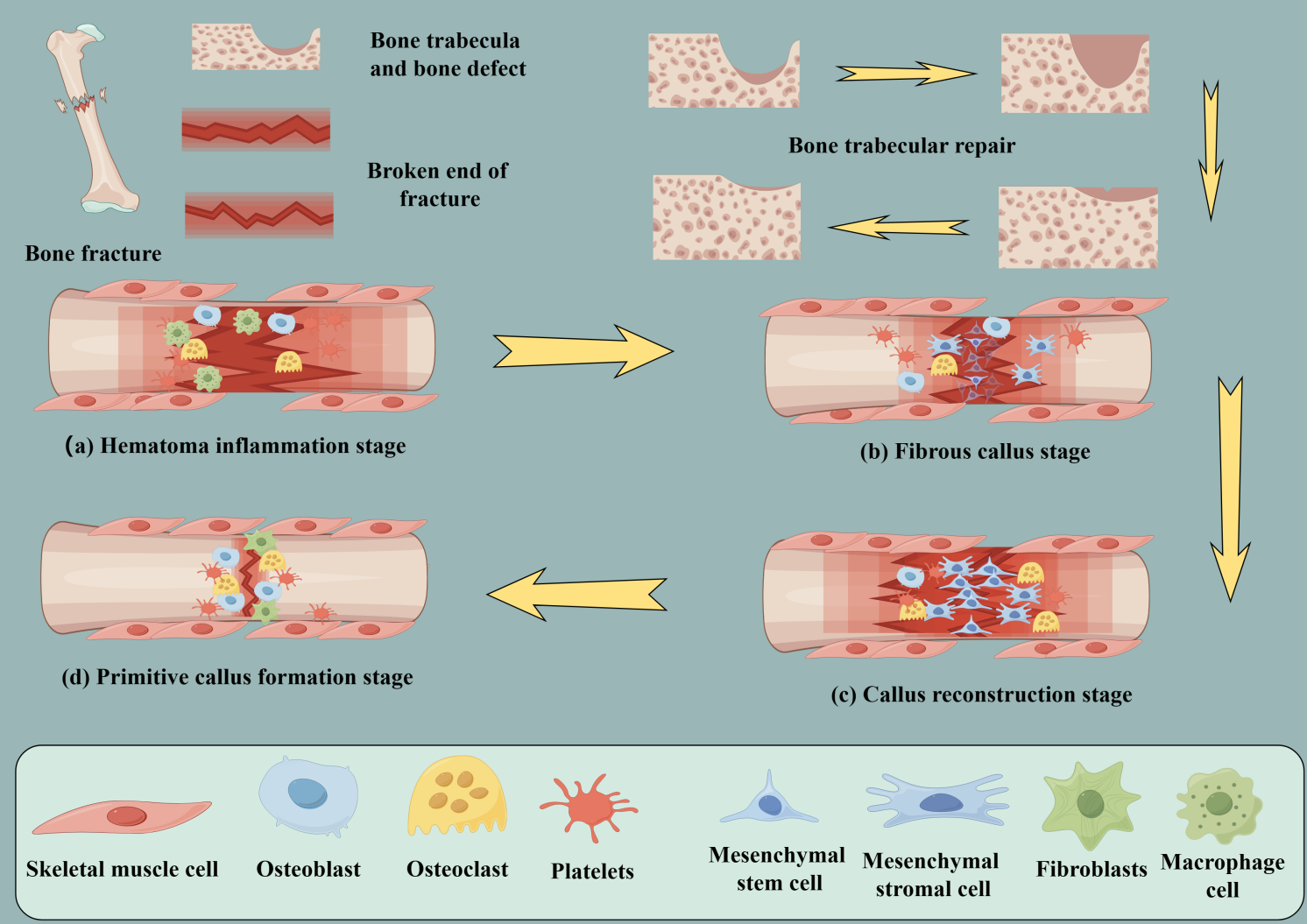


**Figure 7** Schematic diagram of fracture repair and cellular changes; trabecular recovery process.(a) Hematoma inflammation stage (b) Fibrous callus stage (c) Primitive callus formation stage (d) Callus reconstruction stage (by Figdarw).


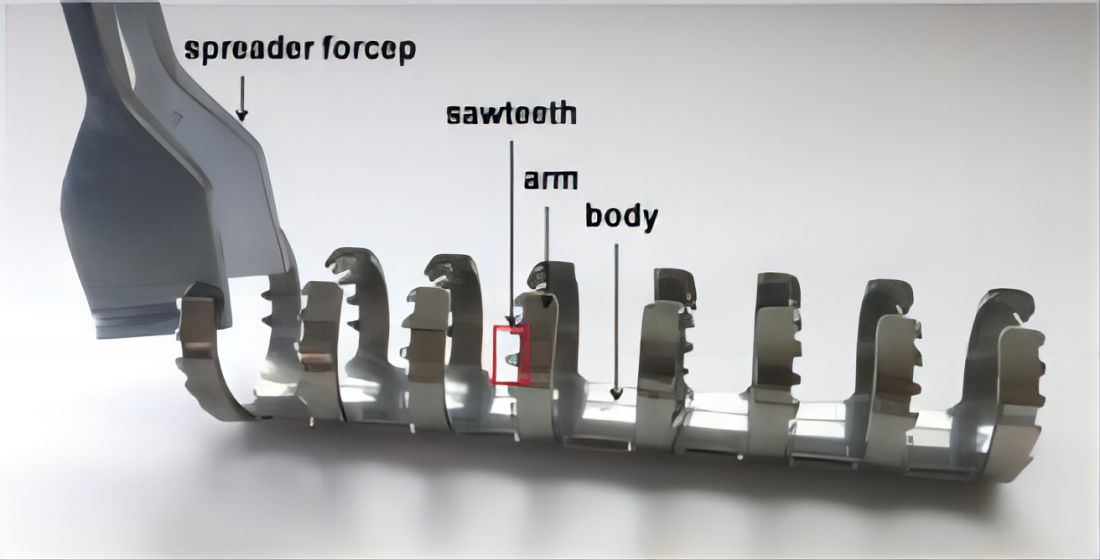


**Figure 8**.The Ni-Ti shape-memory sawtooth-arm embracing clamp (Ni-Ti SSEC) is composed of a body, two arms and a sawtooth. In the axial direction, the arms are symmetrically designed on both sides of the osteotomy. At 0°C-4°C, the arms of the clip are extended to be used with special expansion pliers. When heated with hot brine (40°C-50°C), the arm returns to its original shape and causes Niti SSEC to firmly lock the end of the fracture.

| Porosity (%)  Young’s modulus (GPa)  Critical stress for plastic deformation (MPa) | 0 | 32 | 45 | 58 |
| --- | --- | --- | --- | --- |
|  | 47 | 18 | 13 | 9 |
|  | 1224 | 503 | 398 | 300 |

**Table 1** Variation of elastic modulus with porosity of Ni-Ti alloy at body temperature

| **Categories** | **Main methods or substances** | **Species of cell** | **Osseointegration function** | **References** |
| --- | --- | --- | --- | --- |
| Metal element coating | ZnO nanocoating | Staphylococcus aureus, Staphylococcus pyogenes and Escherichia coli | antibacterial activity↑  frictional forces↓ | ^[29]^ |
|  | Titanium nitride (TiN) coating |  | corrosion resistance↑  frictional forces↓ | ^[30]^ |
|  | Nanocrystalline Ti(OxNy)_2_ (rutile) surface layer | Coliform bacteria | corrosion resistance↑ | ^[31]^ |
|  | RFCVD process  TiO_2_ and a-C: N:H+TiO2 composite layers | platelet | wettability and surface free energy↑platelet adhesion↑Ni2+ release↓ | ^[32]^ |
|  | Ag-SiO2 nano coating |  | Biological activity↑ | ^[33]^ |
|  | Ni43.5Ti45.5W11 (at. %) NiTiW shape memory alloy | Platelet-rich plasma (PRP) ，Murine fibroblast cells (L929) and Human Osteosarcoma cells (MG63) | X-ray radiopacity↑corrosion resistance↑biocompatibility↑ | ^[34]^ |
|  | Silver coating | L. acidophilus | antibacterial activity↑ | ^[35]^ |
|  | Ti‒Ni‒Cu | L929 and MG63  Staphylococcus aureus, Escherichia coli | antibacterial activity↑corrosion resistance↑biocompatibility↑ | ^[36]^ |
| Polymer Coating | Nickel-titanium-oxygen nanopores | pre-osteoblastic cells  Staphylococcus aureus | corrosion resistance↑, Ni2+ release↓cytocompatibility and antibacterial ability↑ | ^[37]^ |
|  | Rhombohedral NiTiO3 nanosheets | Osteoblast, penicillin and streptomycin | corrosion resistance and cytocompatibility↑Ni2+ release↓ | ^[38]^ |
|  | Au @ LDH/B  layered double hydroxides (LDHs) | The cholangiocarcinoma cell line RBE | Anti-tumor activity↑ | ^[39]^ |
|  | LDH/Butyrate | Staphylococcus aureus and Escherichia coli  The cholangiocarcinoma cell line RBE, hepatoma carcinoma cell line SMMC-7721 and breast cancer cell line MCF-7 | Anti-tumor activity↑antibacterial activity↑  Ni2+ release↓ | ^[40]^ |
|  | Ni (OH)_2_ and Ni−Ti LDH | The hepatoma carcinoma cell lines SMMC7721 and HepG2, cholangiocarcinoma cell line RBE, human intrahepatic biliary epithelial cells (HIBEpic) | Anti-tumor activity↑ | ^[41]^ |
|  | Ag nanoparticles (AgNPs)/polylactic acid (PLA)/Al2O3 | Staphylococcus aureus murine fibroblasts (L929) | thermal insulation performance↑ anti­bacterial activity↑biocompatibility↑ | ^[42]^ |
| Nano-scale coating | NiTi crystalline-amorphous nanocomposite (NiTi CAN) |  | fatigue resistance↑ | ^[43]^ |
|  | ultrasonic nanocrystal surface modification (UNSM) | Millipore's Actin Cytoskeleton | biocompatibility↑ corrosion resistance↑ | ^[44]^ |
| Oxidation Coating | Titanium oxide |  | corrosion resistance↑ | ^[45]^ |

**Table 2** Recent advancements in the application of osteogenic coatings on Ni-Ti implants.

| **Category** | **Number of samples** | **Follow-up time** | **Therapeutic effect or**  **Research results** | **References** |
| --- | --- | --- | --- | --- |
| Four-Corner Arthrodesis Concentrator of Ti-Ni Memory Alloy | 18 | 30 months (range, 12–48 months) | Effectively treat the wrist collapse and retain most of the wrist function | ^[98]^ |
| Nickel–titanium (Ni–Ti) memory alloy arthrodesis concentrator | 24 | 12 months (range, 6–24 months) | The Ni–Ti memory alloy arthrodesis concentrator is a convenient tool for scapho-trapezio-trapezoeid (STT) arthrodesis with excellent and reliable results. | ^[99]^ |
| Ti-Ni olecranon memory connector (OMC) | 20 | 3.2 years (range 2-5 years) | The OMC could be an effective alternative to treat olecranon fractures | ^[100]^ |
| Ti-Ni shape-memory sawtooth-arm embracing fixator. | 21 | 39.7 months (range, 1–78 months) | The embracing fixator is a valid alternative treatment for Vancouver type B1 or type C periprosthetic femoral fractures | ^[101]^ |
| Claw-like Ti-Ni SMA fixator (SMA-claw) | 29 | 11.48 months | Ti-Ni SMA claw fixator has good bone grafting effect and can restore stress sustainably. it can replace the traditional tension band technique in the treatment of transverse patellar fracture. | ^[102]^ |
| Ti-Ni shape-memory sawtooth-arm embracing clamp (Ni-Ti SSEC) | 21 | 48.2 months | The Ni-Ti SSEC is a simple and valid method for fixing osteotomies in treating complex femoral revision surgery. | ^[103]^ |
| Ni-Ti arched shape-memory connector (ASC) combined with partially threaded cancellous screws (PTCS) | 21 | 65 months (range, 22-90 months) | ASC combined with PTCS can be used as an effective method for the treatment of supracondylar comminuted fracture of femur. | ^[104]^ |
| Ti-Ni arched shape-memory connector（ASC） | 108 | 42 months (range, 31-53 months) | The ASCs can effectively reduce the incidence of internal fixation loosening, fracture, infection and other complications | ^[105]^ |
| Ti-Ni shape-memory patella concentrator (TNSMPC) | 54 | 12 months | TNSMPC combined with cannulated compression screw is an effective internal fixation method for the treatment of C2 and C3 patellar fractures without additional technical difficulty and tissue injury. | ^[106]^ |
| Ti-Ni arched shape-memory alloy connector | 18 | 4.2 months (range 12–36 weeks) | Nickel-titanium SMA arch connector combined with autogenous bone graft can be used to treat scaphoid nonunion, which is worth popularizing. | ^[107]^ |

**Table 3** The name, sample number, follow-up time and therapeutic effect of nickel-titanium implants used in clinic**.**
